# Lutein Esterification in Wheat Flour Increases the Carotenoid Retention and Is Induced by Storage Temperatures

**DOI:** 10.3390/foods6120111

**Published:** 2017-12-11

**Authors:** Elena Mellado-Ortega, Dámaso Hornero-Méndez

**Affiliations:** Chemistry and Biochemistry of Pigments Group, Food Phytochemistry Department, Instituto de la Grasa (CSIC), Campus Universidad Pablo de Olavide, Ctra. de Utrera km. 1, 41013 Seville, Spain; melladoortegae@gmail.com

**Keywords:** carotenoids, lutein esters, tritordeum, *Triticum turgidum* conv. *durum*, carotenoid retention, whole-grain flour

## Abstract

The present study aimed to evaluate the effects of long-term storage on the carotenoid pigments present in whole-grain flours prepared from durum wheat and tritordeum. As expected, higher storage temperatures showed a catabolic effect, which was very marked for free carotenoid pigments. Surprisingly, for both cereal genotypes, the thermal conditions favoured the synthesis of lutein esters, leading to an enhanced stability, slower degradation, and, subsequently, a greater carotenoid retention. The putative involvement of lipase enzymes in lutein esterification in flours is discussed, particularly regarding the preferential esterification of the hydroxyl group with linoleic acid at the 3′ in the ε-ring of the lutein molecule. The negative effects of processing on carotenoid retention were less pronounced in durum wheat flours, which could be due to an increased esterifying activity (the de novo formation of diesterified xanthophylls was observed). Moreover, clear differences were observed for tritordeum depending on whether the lutein was in a free or esterified state. For instance, lutein-3′-*O*-monolinoleate showed a three-fold lower degradation rate than free lutein at 37 °C. In view of our results, we advise that the biofortification research aimed at increasing the carotenoid contents in cereals should be based on the selection of varieties with an enhanced content of esterified xanthophylls.

## 1. Introduction

Carotenoids, the most widespread pigments in nature, are liposoluble antioxidants produced by plants, algae, fungi, and some bacteria. In plants, carotenoids contribute to the photosynthetic process by acting as light collectors and photoprotectors [1]. Moreover, carotenoids are found in high concentrations in most fruits and flowers where they contribute to the bright colours that attract animals for seed and pollen dispersion. These ubiquitous pigments can also be found in some roots, tubers, and grains, mostly due to the selection of coloured varieties as desired traits for plant domestication by man [2]. Animals are not able to synthetize carotenoids de novo; consequently carotenoids must be acquired through dietary consumption. 

Different carotenoids are associated with different health benefits. The provitamin A activity of carotenoids with at least one unsubstituted β-ring end-group in their structure is well-known and of nutritional significance [3]. Moreover, epidemiological studies have correlated carotenoid intake with protection against a range of chronic diseases, such as cardiovascular diseases and cancer [4,5]. In particular, lutein and zeaxanthin play important roles in the prevention of eye diseases such as age related macular degeneration (AMD), cataracts, and retinitis pigmentosa [6].

Wheat (*Triticum* spp.), one of the most important crops for human consumption worldwide, contains low carotenoid contents compared to most vegetables and fruits. However, the widespread and daily-based consumption of cereals and derived products makes these staple foods an important source of these antioxidants in the diet, particularly in disadvantaged populations. The main objective of biofortification programs is the breeding of crops for better nutrition. The major carotenoid present in wheat is lutein [7]. Among the different *Triticum* species, durum wheat (*Triticum turgidum* ssp. *durum*) grains are characterized for presenting a yellowish colour due to carotenoids. In fact the yellow colour of pasta is a major quality trait [8]. Manipulating the carotenoid content of several cereals, or other crops, by means of biofortification strategies has the potential to provide significant health benefits without altering normal dietetic habits [9]. Remarkable progress has been made in this area; a good example is HarvestPlus, part of the Consultative Group on International Agriculture Research (CGIAR) Program on Agriculture for Nutrition and Health (A4NH). HarvestPlus collaborators have developed staple crops with increased densities of micronutrients through plant breeding techniques, such as pro-vitamin A cassava, which provides up to 40% of the daily vitamin A requirement, iron pearl millet, which provides up to 80% of daily iron needs, and zinc rice, which provides up to 60% of the daily zinc requirement (http://www.harvestplus.org/).

The *Triticeae* tribe, which includes wheat, barley (*Hordeum vulgare*), and rye (*Secale cereale*) species, is a series of closely related polyploids. Fertile amphiploid hybrids can be generated among the different cultivated members of this tribe and some wild cereal species. Tritordeum (*Tritordeum*; 2n = 6x = 42, AABBH^ch^H^ch^) is a cereal obtained from the cross between a wild barley (*Hordeum chilense* Roem. & Schult.) with diploid genome (H^ch^H^ch^) and durum wheat (*Triticum turgidum* conv. *durum*; [10]). The lutein content in tritordeum is about 5–8 times higher than durum wheat and is characterized by a specific esterification profile involving two major fatty acids (linoleic and palmitic acids) [11,12]. The latter characteristic is derived from the genetic background of *H. chilense* [13]. The detailed composition of the lutein esters present in tritordeum has been determined and consists of four monoesters (lutein 3′-*O*-linoleate, lutein 3-*O*-linoleate, lutein 3′-*O*-palmitate, and lutein 3-*O*-palmitate) and four diesters (lutein dilinoleate, lutein 3′-*O*-linoleate-3-*O*-palmitate, lutein 3′-*O*-palmitate-3-*O*-linoleate, and lutein dipalmitate) [12]. Tritordeum is currently the subject of an intense breeding program at the Institute of Sustainable Agriculture (IAS; http://www.ias.csic.es/en/) in Cordoba, Spain, to optimize its use as a cereal for incorporation into the formulation of both functional and novel foods.

Cereal grains are traditionally processed for human consumption. The influence of processing techniques on the composition of phytonutrients and bioactive compounds of many staple foods, including cereal grains, has been extensively studied. For example, in cassava, maize, and sweet potato, the conditions and duration of storage have a more significant negative impact on the retention of provitamin A carotenoids than drying or cooking [14]. Similarly, Mugode et al. [15] concluded that the degradation of provitamin A carotenoids in maize mostly occurred during storage and this effect varied among genotypes. Whole-grain wheat flour contains substantially more vitamins, minerals, antioxidants, and other nutrients, including carotenoids, than refined wheat flour [16]. In addition to milling, the subsequent storage of grains and flours can have a significant impact on the composition of phytochemicals. In fact, cereal flour is usually stored during prolonged periods as part of their industrial and technological treatments, and, therefore, an important impact on the carotenoid content is expected as a result of storage. In the case of wheat flour, the main cause of carotenoid degradation is oxidation (including both enzymatic and non-enzymatic processes) during the storage period. Oxygen present in the medium is considered to be the major factor affecting the stability of carotenoids [17]. Therefore, in addition to considering the “high pigment content” trait of *Triticeae* genotypes, the “carotenoid retention ability” should also be considered when screening and selecting strains for their inclusion in breeding programs [18]. Moreover, new technological treatments are emerging for the processing of cereals and their derived products in order to preserve and enhance the content of carotenoids and other phytonutrients of nutritional relevance (reviewed by Hemery et al. [19]).

The natural process by which xanthophylls are esterified with fatty acids is an important part of post-carotenogenic metabolism and mediates their accumulation in plants. To assist in the development of carotenoid-enhanced cereals, the biochemical characterization of the xanthophyll esterification process and studies of the capacity of the cereal endosperm tissue to store these pigments are necessary. Some studies have highlighted the importance of the carotenoid retention capacity, and its influence on the stability during the postharvest storage of crops [20,21]. However, only a few studies investigated the involvement of xanthophyll esterification in the retention of carotenoids during the storage of cereals and derived products [22,23]. 

We recently assessed the effect of long-term storage on the biosynthesis of lutein esters in durum wheat and tritordeum grains and found that xanthophyll esterification was induced by environmental conditions (especially the temperature) [24]. We also found the xanthophyll esterification process to be highly specific (with the preferential esterification of lutein at position 3 of the β-end ring) and that the fatty acids involved in the esterification and their position in the lutein molecule had a significant effect on the carotenoid stability. Although the results from our previous study increased our understanding of the effects of long-term storage on carotenoid metabolism, characterization of the xanthophyll esterification process was lacking. Therefore, the main goal of the present study was to fully categorize the stability of carotenoid pigments in cereal flours and the influence of pigment esterification during the long-term storage of whole-grain flours in different temperature-controlled conditions. 

## 2. Materials and Methods

### 2.1. Plant Material, Sample Preparation and Storage Conditions

A commercial durum wheat variety (Don Pedro) and a high-carotenoid tritordeum line (HT621, germplasm line developed in the framework of the Cereal Breeding Program carried out at the Institute for Sustainable Agriculture, Córdoba, Spain) [25] were used in the present study. Both samples are considered representatives of these two cereal genotypes and have been previously characterized regarding their carotenoid profile [11,12,23,26]. Plants were grown in 1-L pots, until maturity, under greenhouse conditions with supplementary lights providing a day/night regime of 12/12 h at 22/16 °C. Immediately after harvesting, seeds were preserved for 2 months at 4 °C in a desiccator before the beginning of experiment. After this storage period, and for each cereal genotype, whole-grain flour was obtained from 500 g of grains by using an oscillating ball mill Retsch Model MM400 (Retsch, Haan, Germany) at 25 Hz for 1 min. Subsequently, the resulting flour was distributed in lots of approximately 4 g in round-capped polypropylene 15-mL centrifuge tubes. Flour samples were stored under controlled temperature conditions (−32, 6, 20, 37 and 50 °C) for a period of 12 months. A control sample (t = 0 days) consisting of 5 subsamples was taken and analysed for each cereal type. Triplicate samples (three tubes for each temperature and time) were taken at monthly intervals and analysed in duplicate. The dry matter content (%) in the samples at each sampling date was measured in triplicate by using an Ohaus moisture balance model MB35 (Ohaus, Greifensee, Switzerland). During the course of the experience a continuous monitoring of the storage temperature was performed.

### 2.2. Chemicals and Reagents

Deionised water (HPLC-grade) was produced with a Milli-Q Advantage A10 system (Merck Millipore, Madrid, Spain). HPLC-grade acetone was supplied by BDH Prolabo (VWR International Eurolab, S.L., Barcelona, Spain). The rest of reagents were all of analytical grade.

### 2.3. Extraction of Carotenoids

Carotenoid pigments were extracted from flours with the following procedure. Briefly, 1 g of flour was placed into 25 mL stainless-steel grinding jar together with two stainless-steel balls (15 mm ø), 6 mL of acetone containing 0.1% (w/v) BHT and a known amount of internal standard (β-apo-8′-carotenal; 1.75 and 3.50 μg for durum wheat and tritordeum samples, respectively). Samples were crushed in an oscillating ball mill Retsch Model MM400 (Retsch, Haan, Germany) at 25 Hz for 1 min. Most of the resulting slurry was transferred into a tube and centrifuged at 4500× *g* for 5 min at 4 °C and the clear supernatant collected in a clean tube. The solvent was gently evaporated under a nitrogen stream, and the pigments were dissolved in 0.5 mL of acetone. Prior to the chromatographic analysis, samples were centrifuged at 13,000× *g* for 5 min at 4 °C. The analyses were carried out in duplicate for each sample. All operations were performed under dimmed light to prevent isomerization and photo-degradation of carotenoids.

### 2.4. HPLC Analysis of Carotenoids

The procedures for the isolation and identification of carotenoid pigments and its esters have already been described in previous works [11,12]. Quantitative analysis of carotenoids was carried out by HPLC according to Atienza et al. [11]. The HPLC system consisted of a Waters e2695 Alliance chromatograph fitted with a Waters 2998 photodiode array detector, and controlled with Empower2 software (Waters Cromatografía, S.A., Barcelona, Spain). A reversed-phase column (Mediterranea SEA18, 3 μm, 20 × 0.46 cm; Teknokroma, Barcelona, Spain) was used. Separation was achieved by a binary-gradient elution using an initial composition of 75% acetone and 25% deionized water, which was increased linearly to 95% acetone in 10 min, then raised to 100% in 2 min, and maintained constant for 10 min. Initial conditions were reached in 5 min. An injection volume of 10 μL and a flow rate of 1 mL/min were used. Detection was performed at 450 nm, and the UV-visible spectra were acquired online (350–700 nm wavelength range). Quantification was carried out using calibration curves prepared with lutein, zeaxanthin and β-carotene standards isolated and purified from natural sources [27]. Calibration curves including eight-points were prepared in the pigment concentration range of 0.5–45 μg/mL. Lutein ester content were estimated by using the calibration curve for free lutein, since the esterification of xanthophylls with fatty acids does not modify the chromophore properties [28]. Accordingly, the concentration of lutein esters was expressed as free lutein equivalents. The calibration curve of free lutein was also used to determine the concentration of the (*Z*)-isomers of lutein. Data were expressed as μg/g dry weight (μg/g dw).

### 2.5. Degradation Kinetics Model

In order to investigate the effects of the esterification on the carotenoid degradation during the storage period, the reaction order and derived kinetic parameters were only investigated in those time ranges where the occurrence of catabolic reactions was dominant for each pigment (that is a decline in the concentration with time was clearly observed). For this purpose zero- and first-order kinetics were hypothesized. The general reaction rate expression was applied, −d*C*/d*t* = *k*C*^n^*, where *C* is the concentration of the compound (µg/g dw), *k* is the reaction rate constant (months^−1^), *t* is the reaction time (months), and *n* is the order of the reaction [29]. The reaction rate expression and the kinetic parameters for zero- and first-order models are summarized in Table 1. The selected order of the reaction was that showing the best correlation (*R*^2^) and the best correspondence among the experimental values and the half-life of the compound (t_1/2_) and D (t_1/10_) [time needed for the concentration of a reactant to fall to half and one tenth its initial value respectively, where t_1/2_ = *C*_0_/2*k* and t_1/10_ = 0.9*C*_0_/*k* for zero-order and t_1/2_ = (Ln2)/*k* and t_1/10_ = (Ln10)/*k* for first-order]. Kinetic parameters derived from fitted models with *R*^2^ < 0.8 were not considered in the discussion.

### 2.6. Statistical Analysis

Pigment contents are expressed as mean and standard error of the mean (SEM). Significant differences between means was determined by one-way ANOVA, followed by a post-hoc test of mean comparison using the Duncan test for a confidence level of 95% (*p* < 0.05) utilizing the STATISTICA 6.0 software (StatSoft Inc., Tulsa, OK, USA). 

## 3. Results and Discussion

### 3.1. Carotenoid Content in Whole-Grain Flours: Effect of Long-Term Storage

The initial carotenoid composition for the flours of durum wheat (*Triticum turgidum* conv. *durum*, Don Pedro) and tritordeum (Tritordeum HT621 line) was consistent with previous studies (see Table 2 at t = 0 months) [11,12,23,26]. The initial concentrations of individual pigments were higher in whole-grain tritordeum flour than durum wheat flour, with the exception of (all-*E*)-zeaxanthin, which is not present in tritordeum. On average, the total initial carotenoid content was six times higher in tritordeum (HT621 line) with respect to durum wheat (Don Pedro). 

**Figure 1:**
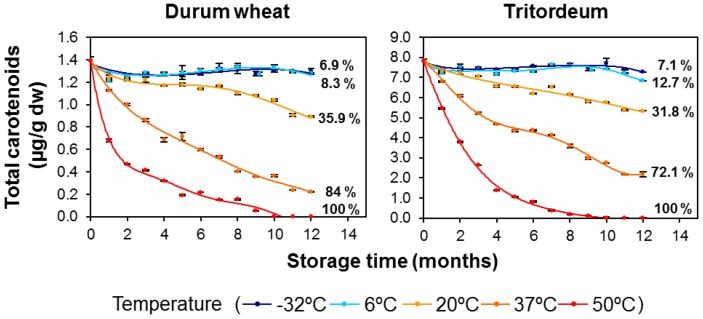
Evolution of the total carotenoid content (μg/g dry weight) in durum wheat (Don Pedro variety) and tritordeum (HT621 advanced line) whole-grain flours during long-term storage under temperature controlled conditions (−32, 6, 20, 37 and 50 °C). The values shown are the mean and standard error (*n* = 5 for the starting sample, *n* = 3 for the rest of the samples). Pigment losses (%) are indicated at the end of storage.

The evolution of total carotenoids revealed significant losses for both cereals at the higher examined temperatures of 37 and 50 °C (Figure 1; see also Figure S1, and Tables S1 and S2). The total degradation of pigments was observed by the end of the 12-month storage period in the durum wheat and tritordeum samples kept at the higher temperature (50 °C). In accordance with Gayen et al. [30], the experimental conditions tested in the present study may facilitate the action of degradative enzymes, such as lipoxygenase (LOX), leading to the co-oxidation of carotenoid pigments. LOX is mostly located in the germ and bran of the grain kernel and its main substrate is linoleic acid [31]. In addition to LOX degradation, the susceptibility of carotenoids to oxygen and high temperatures should be responsible for the decrease in the pigment levels observed during our experiments. The pigment content reduction at the end of the 12-month storage period at −32, 6, and 20 °C were similar in all cases for both cereal genotypes. In contrast, significant differences were observed between the two cereals at 37 °C, with a greater retention of pigments observed in tritordeum (pigment losses of 84% for durum wheat compared to 72% for tritordeum) (Figure 1). 

**Figure 2:**
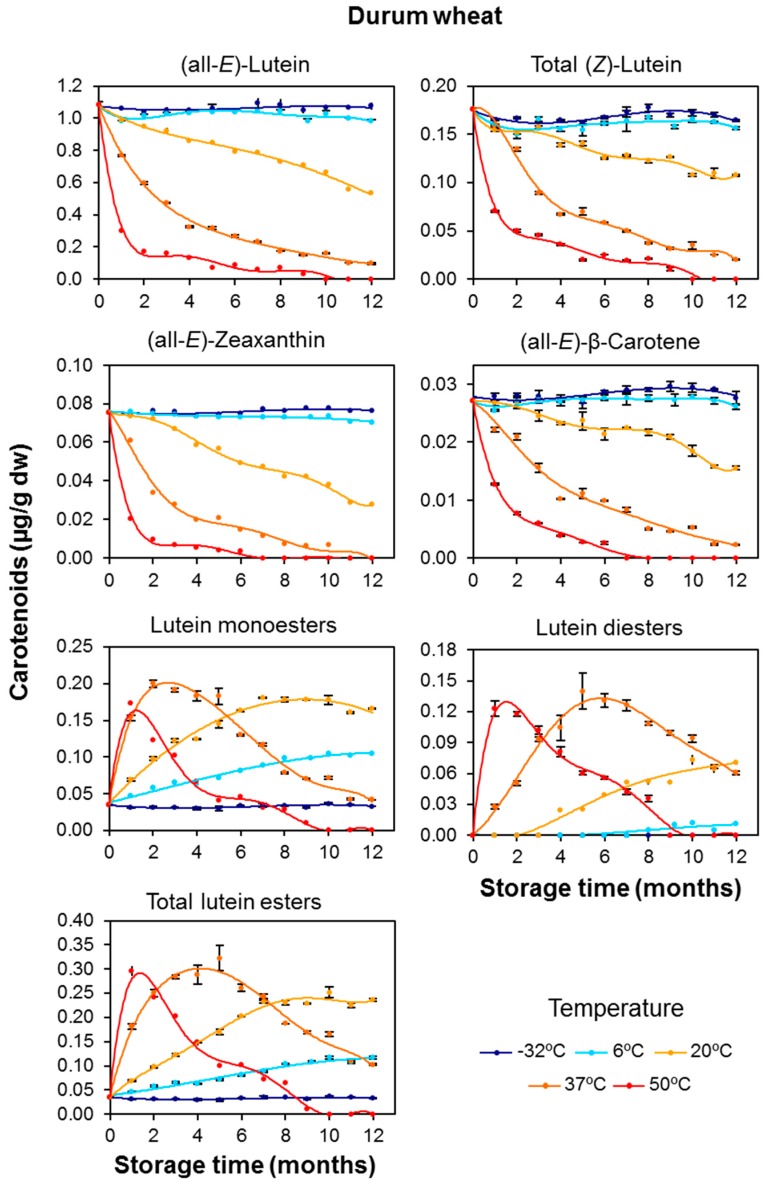
Evolution of the individual carotenoid content and esterified fractions (μg/g dry weight) in durum wheat (Don Pedro variety) whole-grain flours during long-term storage under temperature controlled conditions (−32, 6, 20, 37 and 50 °C). The values shown are the mean and standard error (*n* = 5 for the starting sample, *n* = 3 for the rest of the samples).

The changes observed for the individual free pigments in durum wheat (Figure 2) were similar to the trend described for total carotenoid content: The carotenoid content remained fairly constant at the lowest storage temperatures of −32 and 6 °C while higher carotenoid losses were observed with increased storage temperature. Thus, the carotenoid losses after 12 months of storage at 20 °C were 51% and 63% for (all-*E*)-lutein and (all-*E*)-zeaxanthin, respectively (Table S1). The declines were lower at 20 and 37 °C for the (*Z*)-isomers of lutein (39% and 89%, respectively), which is consistent with a *trans* to *cis* isomerization process, as reported in other similar studies [32]. At the end of the storage period at 37 °C, (all-*E*)-lutein, (all-*E*)-zeaxanthin, and (all-*E*)-β-carotene had been degraded to trace levels. For the durum wheat flour maintained at 50 °C, (all-*E*)-zeaxanthin and (all-*E*)-β-carotene were already undetectable at the seventh month. These results suggest that both zeaxanthin and β-carotene are less thermostable than lutein at higher temperatures. Some authors have suggested that (all-*E*)-β-carotene is the most thermolabile carotenoid, especially in low-water environments as in the case of cereal flour [33].

**Figure 3:**
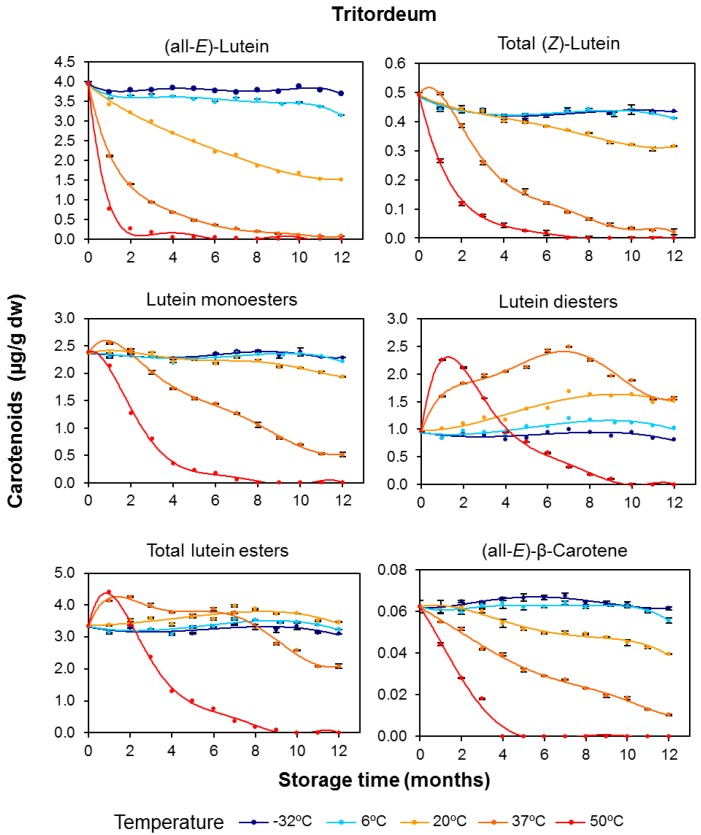
Evolution of the individual carotenoid content and esterified fractions (μg/g dry weight) in tritordeum (HT621 advanced line) whole-grain flours during long-term storage under temperature controlled conditions (−32, 6, 20, 37 and 50 °C). The values shown are the mean and standard error (*n* = 5 for the starting sample, *n* = 3 for the rest of the samples).

For whole-grain tritordeum flour, greater losses of the individual free pigments were also observed at the higher storage temperatures (Figure 3). For tritordeum flour stored at 37 °C, (all-*E*)-lutein, (*Z*)-lutein, and (all-*E*)-β-carotene had decreased by 83–98% by the end of the 12-month storage period (Table S2). Similar to durum wheat, the total pigments in tritordeum were completely destroyed after 10 months of storage at 50 °C, and (all-*E*)-β-carotene was already undetectable after 4 months.

In contrast to the free carotenoids, the lutein esters showed increased levels with increasing temperature. This distinction was particularly striking for durum wheat, and especially the monoester fraction, stored at the milder conditions of 6 and 20 °C (Figure 2). For example, the concentration of lutein monoesters increased by 3.7 and 5.7 times the initial values after 12 months at 6 and 20 °C, respectively. At the higher temperatures (37 and 50 °C), competition and/or compensation was observed between the increases promoted by the temperature (de novo esterification) and decreases caused by oxidative degradation. After the storage of durum wheat for 12 months, concentration increases of 2–3 fold were recorded at 37 °C for the lutein ester fraction (sum of monoesters and diesters), revealing an intense esterifying activity under these conditions. The concomitant formation of lutein diesters associated with the rise in storage temperature and time was prompted by the increase in the pool of monoesters.

The free pigments in tritordeum showed a general pattern of degradation with increasing temperature; in contrast, analysis of the esterified fractions in tritordeum revealed clear differences in the evolution profile of lutein monoesters and diesters (Figure 3). Similar to the free pigments, the monoesters fraction decreased by 18.4% and 78.3% with respect to initial levels after 12-months storage at 20 and 37 °C, respectively. However, the diester fraction showed a similar behaviour to that found in durum wheat, with increases of 7%, 60%, and 63% with respect to initial levels after 12-months storage at 6, 20, and 37 °C, respectively. These results might indicate either the occurrence of different isoforms of the esterifying enzymes in the two cereals, or a more intensive esterifying activity of the enzymes in durum wheat. The *H. chilense* genetic background of tritordeum could be responsible for these differences. 

The synthesis of lutein esters in cereal flours could possibly take place through a different metabolic pathway from the one operating in intact grains. In fact, in a preliminary study carried out with durum wheat flour [23], the formation of lutein esters was observed, especially at high temperatures; however, the trace levels of the pigments impaired their quantification. The present results underline the importance of controlling the storage conditions of cereals and derived products in order to prevent or promote changes in the profile of carotenoids and other phytochemicals. One possibility is that the esterification of xanthophylls in flours is mediated by the activity of lipases, which are concentrated in the bran in the case of cereals [34]. Lipases catalyse the hydrolysis of carboxyl-ester linkages leading to the release of fatty acids and organic alcohols. However, under low-water conditions, lipases may catalyse the reverse reaction (esterification) or various transesterification reactions involving acids, alcohols, and esters [35]. The acyl transferase activity of lipases has already been suggested to be involved in the formation of sterol esters during the long-term storage of wheat flour [36]. These authors also observed the esterification of lutein, although the data obtained were inconclusive. More recently, Ahmad et al. [37] have provided sound data concluding that lutein esterification in bread wheat was genetically controlled and likely due to a GDSL-lipase located on the 7D chromosome and co-located with a QTL (Quality Trait Loci) associated with ester formation. Moreover, Mattera et al. [38,39] demonstrated that lutein esterification in wheat endosperm is controlled by chromosomes of the homoeologous group 7 (7D and 7H^ch^), and suggested differential fatty acid enzyme specificity depending on the cereal species (for instance, common wheat and *H. chilense*).

### 3.2. Effect of Long-Term Storage on the Esterified Lutein Fractions

**Figure 4:**
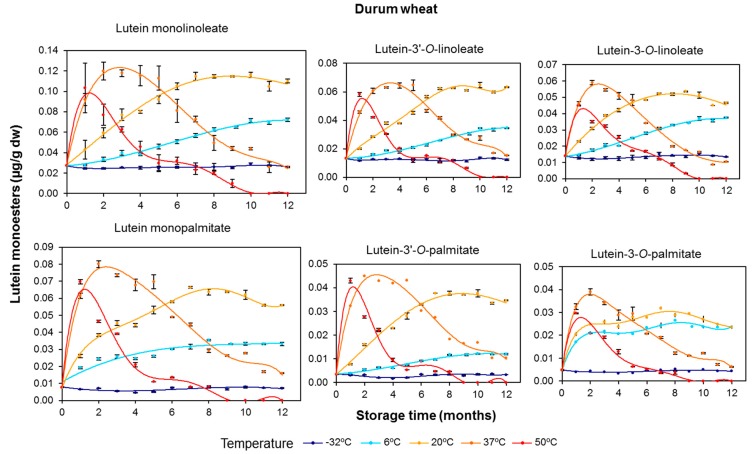
Quantitative changes in the xanthophyll ester fractions (μg/g dry weight) in durum wheat (Don Pedro variety) whole-grain flours during long-term storage under temperature controlled conditions (−32, 6, 20, 37 and 50 °C). The values shown are the mean and standard error (*n* = 5 for the starting sample, *n* = 3 for the rest of the samples).

The evolution of individual lutein monoesters (lutein monolinoleate and monopalmitate) was similar and consistent with the corresponding changes observed for the total esters fraction during the entire storage of both cereal flours. The profiles at 37 and 50 °C presented two distinctive areas of net synthesis (peaks) and degradation (troughs). This was particularly evident in durum wheat (Figure 4). At 37 °C, lutein monolinoleate showed a synthesis period over the first 2 months of storage, with a 4-fold concentration increase, followed by a degradation period during the remaining storage time, reaching up to 78% total degradation. Similarly, at 50 °C, the maximum synthesis was observed after the first month, accounting for concentrations of up to 3 times the initial content. Lutein monopalmitate showed a more pronounced synthesis, with a 10-fold increase observed after two months at 37 °C and an 8-fold increase after the first month at 50 °C (Figure 4). These data indicate differences in relation to the preferential formation of lutein monoesters with both fatty acids, with the palmitic acid esters being more abundant. On the other hand, the degradation rate was similar for lutein monopalmitate and lutein monolinoleate during the storage of durum wheat flour at 37 and 50 °C. Thus, the ratio between lutein monopalmitate and lutein monolinoleate remained constant across the whole storage period (Table S1). However, in a previous study with durum wheat grains submitted to long-term storage, a higher thermostability for lutein monolinoleate was reported [24]. 

**Figure 5:**
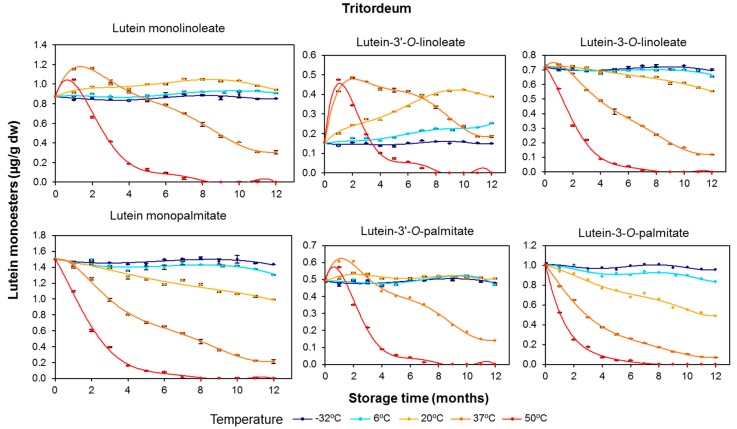
Evolution of the monoesterified lutein (μg/g dry weight), including the regioisomers, in tritordeum (HT621 advanced line) whole-grain flours during long-term storage under temperature controlled conditions (−32, 6, 20, 37 and 50 °C). The values shown are the mean and standard error (*n* = 5 for the starting sample, *n* = 3 for the rest of the samples).

In tritordeum, the maximum concentration increase for lutein monolinoleate was registered after two months at 37 °C and one month at 50 °C (Figure 5). In contrast to durum wheat, stability differences between both monoesters were observed in tritordeum. Lutein monolinoleate remained mostly constant at −32, 6, and 20 °C, whereas lutein monopalmitate showed a progressive decrease with increasing temperature and storage time. Nevertheless, the degradation rates for lutein monoesters were consistently lower than the rates recorded for free lutein (Figure 3). The degradation after 12 months at 37 °C was greater for lutein monopalmitate (85.9%) than for lutein monolinoleate (65.1%).

With respect to the increases in the lutein ester contents observed in durum wheat (Figure 2 and Figure 4), the putative lipase enzyme could exhibit greater activity for palmitic acid than for linoleic acid due to a higher specificity for palmitic acid as a substrate or a better availability of this saturated fatty acid. These results are in line with those obtained by O’Connor et al. [40] who reported the lipase activity in different cereals. However, the evolution of the esterified fraction in tritordeum flour suggested a different scenario compared to durum wheat. In order to interpret this data, it should be taken into consideration that tritordeum is a novel hybrid cereal in which the *H. chilense* genome contributes and interacts with that of durum wheat. Therefore, it is likely that these genetic differences are the cause for the lower esterification activity in tritordeum flour. 

Regarding the two regioisomers for each lutein monoester, the 3′ position of the lutein molecule appears to be the preferred site for esterification mediated by lipases in both cereals (Figure 4 and Figure 5), and gives rise to a more stable compound (i.e., lutein-3′-*O*-linoleate and lutein-3′-*O*-palmitate are more stable than lutein-3-*O*-linoleate and lutein-3-*O*-palmitate, respectively). These observations reinforced the idea that the enzyme systems involved in the esterification of lutein in cereal flours are different from those operating in intact grains [12].

**Figure 6:**
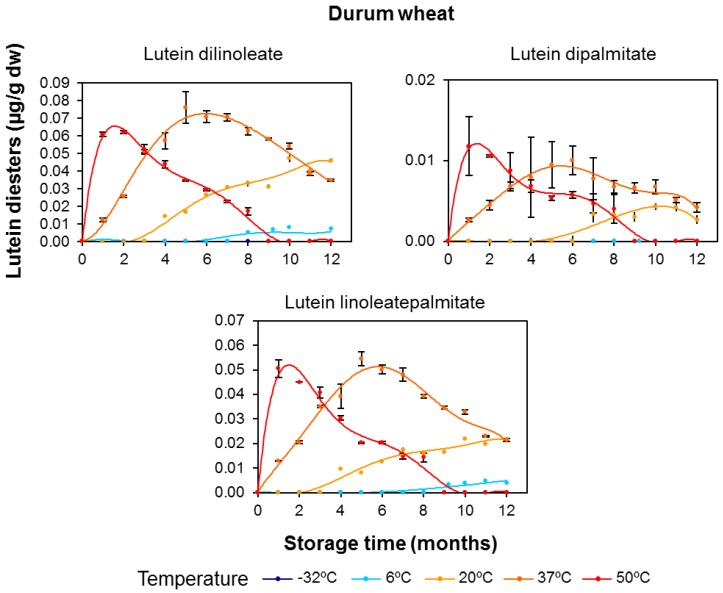
Evolution of the diesterified lutein (μg/g dry weight) in durum wheat (Don Pedro variety) whole-grain flours during long-term storage under temperature controlled conditions (−32, 6, 20, 37 and 50 °C). The values shown are the mean and standard error (*n* = 5 for the starting sample, *n* = 3 for the rest of the samples).

**Figure 7:**
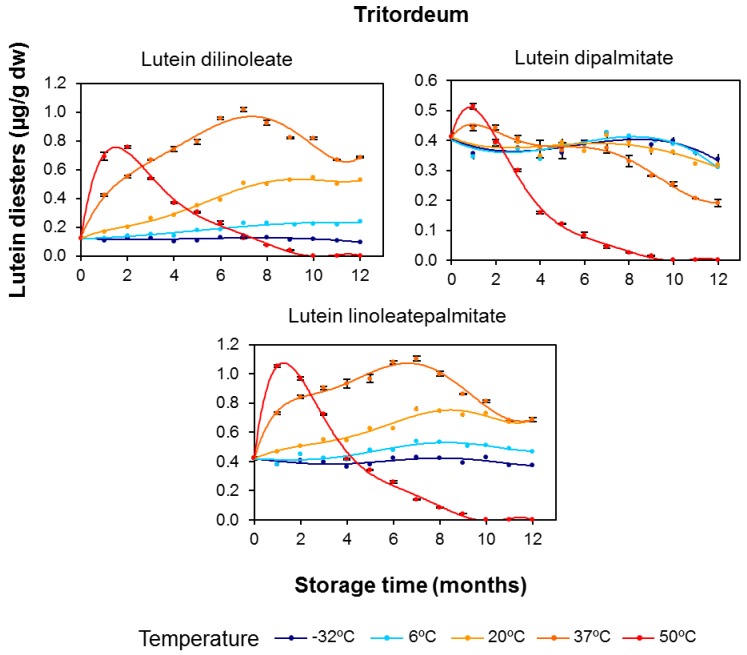
Evolution of the diesterified lutein (μg/g dry weight) in tritordeum (HT621 advanced line) whole-grain flours during long-term storage under temperature controlled conditions (−32, 6, 20, 37 and 50 °C). The values shown are the mean and standard error (*n* = 5 for the starting sample, *n* = 3 for the rest of the samples).

In both cereal flours, lutein diesters presented remarkable net increases during the duration of the storage period, especially for lutein dilinoleate and particularly at the storage temperature of 37 °C (Figure 6 and Figure 7). The evolution of diesterified xanthophylls over the storage period clearly suggests that their formation is due to induction of the esterification process by temperature, with this synthesis being particularly prominent in durum wheat. As the storage temperature increased, the rate and amounts of synthesis were also increased, but, on the other hand, the degradative processes due to oxidative stress were also induced. Interestingly, the higher the degree of esterification (diester > monoester > free), the higher the xanthophyll’s stability and, consequently, the more delayed the degradation (Figure 4 and Figure 6 for durum wheat and Figure 5 and Figure 7 for tritordeum).

### 3.3. Kinetics of Retention of Carotenoids during the Long-Term Storage of Wheat Flours

A kinetic study indicated a progressive increase of the rate constants (*k*) with the rise of temperature for both cereal genotypes. Table 3 and Table 4 summarized the kinetic data characterizing the evolution of both free and total pigments (including esterified pigments) during long-term storage assuming zero- and first-order kinetic models, respectively. As deducted from the correlation coefficient values, the first-order kinetic model showed best adjustment to the data, indicating that the degradation reaction rate is directly proportional to the pigment concentration (−d*C*/d*t* = *kC*; see Materials and Methods and Table 1). These results are consistent with those reported by other authors [32,41]. The reaction rates were higher in tritordeum for all pigments at 20, 37, and 50 °C, with β-carotene being an exception. At 50 °C, the *k* values for all xanthophylls were approximately double in tritordeum compared to durum wheat. Pigment structure (free or esterified with fatty acids), matrix effects (including the presence of oxidative enzymes and other antioxidants), and the oxidative stressing environment are likely to be key factors for this phenomena. The milling process, involving cell and tissue disruption, and the subsequent storage of cereal flours have also previously been found to affect carotenoid stability [42]. The possible presence of other antioxidants might also produce a protective effect on the carotenoids. For example, the changes and/or interactions between tocopherols and carotenoids during cereal processing have been analysed by several authors [43,44]. However, there is no information about such interactions in tritordeum, with further work needed in this area.

The *k* values obtained for durum wheat were similar for xanthophylls and carotenes (β-carotene) with some exceptions (Table 4). (all-*E*)-Zeaxanthin was the pigment that was degraded most rapidly at all temperatures. As expected, the *k* value for zeaxanthin was maximum at 50 °C (473 × 10^−3^ month^−1^), which is consistent with its complete disappearance at the seventh month in these conditions (Figure 2). Markedly, the differences between the *k* values for carotenes and xanthophylls were more evident in tritordeum flour. These results are in line with those obtained by Dhuique-Meyer et al. [45], whose study about the thermal degradation kinetics of vitamin C and carotenoids in citrus juices reported lower degradation rates for β-carotene than for xanthophylls.

The kinetic data obtained for the total carotenoids (which included the esterified pigments) confirmed a faster degradation in tritordeum than in durum wheat, especially at the higher storage temperatures, as indicated by the *k* values at 50 °C (Table 4). In the case of durum wheat flours, the *k* values at 20, 37, and 50 °C were consistently lower for total lutein and total carotenoids (including the xanthophyll esters) than for the respective free fractions, underlining the contribution of the esters to the greater stability of such fractions. Accordingly, this effect was more pronounced in tritordeum due to the higher content and proportion of esterified lutein.

The rate constants for lutein esters, including the distinction between regioisomers, are summarized in Table 5. In line with the results for total carotenoids (Table 4), the esterified fractions showed a higher degradation rate in tritordeum with compared to durum wheat. Notably, the *k* value for lutein diesters at 50 °C in tritordeum was double the *k* value in durum wheat. This result highlights an important turnover of diesters in durum wheat in accordance with a possible esterifying activity in this cereal. The differential content of other pro-oxidant and antioxidant substances in both cereal genotypes should also be considered. In any case, the *k* values decreased with an increase in the degree of esterification, with *k* lutein diester < *k* lutein monoester < *k* free lutein, resulting in an increased thermostability thereof. Regarding the regioisomers of the lutein monoesters (lutein monolinoleate and lutein monopalmitate), both positions 3 and 3′ had similar degradation rates for each lutein monoester. No relevant differences between free and esterified lutein (Table 4 and Table 5) were found in durum wheat, with the exception of the diesters. In tritordeum, lower degradation rates were recorded for lutein monoesters and diesters, even at 50 °C, despite the intense degradative conditions at that temperature. Lutein-3′-*O*-monolinoleate showed a degradation rate approximately 3-fold lower compared to free lutein at 37 °C. Within the monoester fraction, lutein monolinoleate presented a slower degradation than lutein monopalmitate; the same trend was observed for the monoesters at position 3′ compared with the counterpart regioisomer at position 3. These data are consistent with the evolution described for the esterified fractions. In the case of the diesters, the degradation rates were even lower with no differences between the different acylated forms. 

Half-life values (t_1/2_; months) and D values (t_1/10_; months) (Table 4) are a very useful tool for estimating the pigment concentration that will be retained in flours stored under controlled temperature conditions. Both parameters are inversely related to *k* values, so that an increase in the storage temperature results in a reduction in the t_1/2_ and t_1/10_ values. The half-life and D values were generally higher for durum wheat for all pigments at all storage temperatures. Thus, total free lutein and free carotenoids showed longer half-life values by 2 and 1 extra month, and D values of 5 and 4 extra months at 37 °C and 50 °C, respectively, for durum wheat compared to tritordeum. As an exception, the observed half-life and D values at 37 °C for total lutein and total carotenoids revealed the opposite situation, with tritordeum flour having higher values. These results are directly related to the higher content and proportion of esterified pigments in tritordeum, and they are in line with the lower carotenoid losses in tritordeum compared to durum wheat at 37 °C (Figure 1).

## 4. Conclusions

This comparative study evaluated the effects of processing and storage of whole-grain durum wheat and tritordeum flours on the total carotenoid content. The influence of the cereals’ different genetic backgrounds was found to be important and the effect of storage was more severe on the carotenoid content of tritordeum. Tritordeum flour showed a lower retention of free and esterified carotenoids than durum wheat flour. This could be mediated by an increased esterifying activity in durum wheat flours and/or greater oxidative enzymatic activity, or a more oxidative environment in tritordeum flour. These results could be influenced by the fact that durum wheat varieties have generally suffered domestication and selective pressure by man for the preservation of the yellow colour trait. Our results suggest the occurrence of an enzymatic process, maybe a lipase, involved in the esterification of xanthophylls during the storage of these flours. The enzyme showed a preferential action for esterification of the hydroxyl group at position 3′ in the ε-ring of the lutein molecule with linoleic acid. We hypothesize that this process could be different to the one described in intact grains in which the responsible enzymes (XAT: xanthophyll acyltransferase) showed a preferential acylation for the β-ring and a higher selectivity for palmitic acid, and therefore further research needs to be carried out to contrast this hypothesis. In any case, the increase in esterified xanthophylls eventually derived in a higher stability and retention capacity for total carotenoids in both cereal flours. This study provides valuable information to inform the optimization of storage conditions for flours of durum wheat and the novel hybrid cereal tritordeum with the aim of preserving their phytochemicals. This information could also be used in crop biofortification programs for the selection of cereal varieties, such as tritordeum, with an enhanced content of esterified xanthophylls.

## Figures and Tables

**Table 1:** Expression of the reaction rate depending on the reaction order (*n*) and kinetic parameters derived.

Reaction Order	Reaction Rate Expression	Integrated Expression	Graphical Representation	Half-Life ^a^ (t_1/2_)	D ^b^ (t_1/10_)
Zero *n* = 0	−d*C*/d*t* = *kC*^0^ = *k*	*C*-*C*_0_ = −*kt*	*C*-*C*_0_ vs. *t* Slope = −*k*	t_1/2_ = *C*_0_/2*k*	t_1/10_ = 0.9*C*_0_/*k*
First *n* = 1	−d*C*/d*t* = *kC*^1^ = *kC*	Ln(*C*/*C*_0_) = −*kt*	Ln(*C*/*C*_0_) vs. *t* Slope = −*k*	t_1/2_ = Ln(2)/*k*	t_1/10_ = Ln(10)/*k*

^a^ Time needed for the concentration of a reactant to fall to half of its initial value. ^b^ Time needed for the concentration of a reactant to fall one tenth of its initial value.

**Table 2:** Initial carotenoid composition in *Triticum turgidum* cv. *durum* (Don Pedro variety) and *Tritordeum* (HT621 line) whole-grain flours subjected to long-term storage (12 months) under controlled temperature.

HPLC Peak ^a^	Pigment	Concentration (μg/g Dry Weight) ^b^	
		Durum Wheat (Don Pedro Variety)	Tritordeum (HT621 Advanced Line)
1	(all-*E*)-Zeaxanthin	0.08 ± 0.00	-
2	(all-*E*)-Lutein	1.08 ± 0.02	3.95 ± 0.04
3	(9*Z*)-Lutein	0.06 ± 0.00	0.19 ± 0.01
4	(13*Z*)-Lutein	0.12 ± 0.00	0.30 ± 0.01
5	Lutein-3′-*O*-linoleate	0.01 ± 0.00	0.15 ± 0.00
6	Lutein-3-*O*-linoleate	0.01 ± 0.00	0.72 ± 0.01
5 + 6	Lutein monolinoleate	0.03 ± 0.00	0.87 ± 0.00
7	Lutein-3′-*O*-palmitate	0.00 ± 0.00	0.49 ± 0.01
8	Lutein-3-*O*-palmitate	0.00 ± 0.00	1.01 ± 0.02
7 + 8	Lutein monopalmitate	0.01 ± 0.00	1.50 ± 0.01
9	(all-*E*)-β-Carotene	0.02 ± 0.00	0.06 ± 0.00
10	Lutein-3,3′-dilinoleate	n.d. ^c^	0.12 ± 0.00
11	Lutein-3′-*O*-linoleate-3-*O*-palmitate plus Lutein-3′-*O*-palmitate-3-*O*-linoleate	n.d.	0.42 ± 0.01
12	Lutein-3,3′-dipalmitate	n.d.	0.41 ± 0.01
	Lutein monoesters	0.04 ± 0.00	2.37 ± 0.01
	Lutein diesters	-	0.96 ± 0.01
	Total lutein esters	0.04 ± 0.00	3.33 ± 0.02
	Total free lutein	1.26 ± 0.02	4.44 ± 0.09
	Total lutein	1.29 ± 0.02	7.77 ± 0.07
	Total carotenoids	1.39 ± 0.03	7.83 ± 0.07
	**Regioisomers ratios**		
	Lutein-3-*O*-linoleate/Lutein-3′-*O*-linoleate	1	5
	Lutein-3-*O*-palmitate/Lutein-3′-*O*-palmitate	1	2

^a^ Peak numbers according to Figure S1 (Supplementary Materials). ^b^ Data represent the mean ± standard error (*n* = 5). ^c^ n.d. not detected.

**Table 3:** Reaction rate constant (*k*; month^−1^) for the total carotenoid content in durum wheat (Don Pedro variety) and tritordeum (HT621 line) whole-grain flours during a long-term storage period (12 months) at −32, 6, 20, 37, and 50 °C following the zero-order kinetic model (*C*-*C*_0_ = −*kt*).

Pigment	T (°C)	Durum Wheat (Don Pedro Variety)		Tritordeum (HT621 Advanced Line)	
		*k* (×10^−3^ Month^−1^)	*R*^2^	*k* (×10^−3^ Month^−1^)	*R*^2^
(all-*E*)-Zeaxanthin	−32	0.2	0.33	-	-
	6	0.4	0.80	-	-
	20	4	0.98	-	-
	37	5	0.80	-	-
	50	3	0.43	-	-
(all-*E*)-Lutein	−32	1	0.06	7	0.15
	6	3	0.18	41	0.72
	20	42	0.98	198	0.96
	37	68	0.80	232	0.65
	50	51	0.47	160	0.33
Total free (*Z*)-Lutein	−32	0.3	0.04	2	0.14
	6	0.2	0.01	3	0.31
	20	5	0.92	15	0.97
	37	12	0.86	42	0.87
	50	10	0.63	28	0.57
(all-*E*)-β-Carotene	−32	0.1	0.35	0.1	0.02
	6	0.5	0.06	0.2	0.12
	20	8	0.93	2	0.95
	37	2	0.90	4	0.97
	50	1	0.62	4	0.62
Total free lutein	−32	1	0.06	9	0.19
	6	3	0.15	42	0.70
	20	47	0.98	213	0.95
	37	80	0.82	273	0.70
	50	60	0.50	188	0.36
Total free carotenoids	−32	1	0.01	9	0.19
	6	6	0.20	44	0.70
	20	50	0.96	215	0.96
	37	85	0.80	277	0.70
	50	64	0.48	192	0.36
Total lutein	−32	2	0.08	10	0.07
	6	4	0.15	29	0.21
	20	23	0.88	191	0.95
	37	85	0.95	432	0.95
	50	80	0.73	546	0.75
Total carotenoids	−32	0.8	0.01	10	0.07
	6	1	0.01	29	0.21
	20	33	0.88	192	0.95
	37	90	0.94	436	0.95
	50	83	0.71	550	0.75

**Table 4:** Reaction rate constant (*k*; month^−1^), half-life (t_1/2_; months), and D (t_1/10_; months) for the total carotenoid content in durum wheat (Don Pedro variety) and tritordeum (HT621 line) whole-grain flours during a long-term storage period (12 months) at −32, 6, 20, 37, and 50 °C following the first-order kinetic model (Ln(*C*/*C*_0_) = −*kt*).

Pigment	T (°C)	Durum Wheat (Don Pedro Variety)				Tritordeum (HT621 Advanced Line)			
		*k* (×10^−3^ Month^−1^)	*R*^2^	t_1/2_ (Months)	t_1/10_ (D) (Months)	*k* (×10^−3^ Month^−1^)	*R*^2^	t_1/2_ (Months)	t_1/10_ (D) (Months)
(all-*E*)-Zeaxanthin	−32	3	0.33	277	921	-	-	-	-
	6	5	0.79	141	470	-	-	-	-
	20	85	0.96	8	27	-	-	-	-
	37	252	0.97	3	9	-	-	-	-
	50	473	0.87	1	5	-	-	-	-
(all-*E*)-Lutein	−32	1	0.06	693	2302	2	0.15	385	1279
	6	3	0.18	217	719	12	0.72	60	198
	20	53	0.96	13	43	82	0.99	8	28
	37	191	0.97	4	12	329	0.99	2	7
	50	300	0.84	2	8	596	0.88	1	4
Total free (*Z*)-lutein	−32	2	0.04	385	1279	4	0.13	165	548
	6	0.7	0.00	990	3289	6	0.31	110	365
	20	38	0.93	18	60	39	0.98	18	59
	37	177	0.98	4	13	283	0.99	2	8
	50	249	0.88	3	9	559	0.99	1	4
(all-*E*)-β-Carotene	−32	5	0.35	139	460	1	0.02	576	1918
	6	2	0.06	462	1535	3	0.12	210	698
	20	45	0.90	16	52	38	0.95	19	62
	37	203	0.96	3	11	141	0.98	5	16
	50	387	0.95	2	6	417	0.99	2	6
Total free lutein	−32	1	0.07	693	2302	2	0.19	330	1096
	6	3	0.15	248	822	11	0.71	63	209
	20	51	0.97	14	45	76	0.99	9	30
	37	189	0.98	4	12	321	0.99	2	7
	50	290	0.85	2	8	630	0.94	1	4
Total free carotenoids	−32	0.7	0.01	990	3289	2	0.19	347	1151
	6	5	0.20	151	500	11	0.70	64	211
	20	53	0.96	13	44	75	0.99	9	31
	37	191	0.97	4	12	311	0.99	2	7
	50	296	0.85	2	8	636	0.94	1	4
Total lutein	−32	1	0.08	533	1771	1	0.07	495	1644
	6	3	0.15	248	822	4	0.21	178	590
	20	27	0.86	25	84	30	0.96	23	77
	37	145	0.98	5	16	103	0.98	7	22
	50	285	0.93	2	8	465	0.98	1	5
Total carotenoids	−32	0.5	0.00	1386	4604	1	0.07	495	1644
	6	0.9	0.01	770	2558	4	0.21	178	590
	20	30	0.87	23	78	30	0.96	23	77
	37	148	0.98	5	16	103	0.98	7	22
	50	290	0.93	2	8	466	0.98	1	5

**Table 5:** Reaction rate constants (*k*; month^−1^) for the esterified carotenoid content in durum wheat (Don Pedro variety) and tritordeum (HT621 line) whole-grain flours during a long-term storage period (12 months) at 20, 37, and 50 °C following the first-order kinetic model (Ln(*C*/*C*_0_) = −*kt*).

Pigment	T (°C)	Durum Wheat (Don Pedro Variety)		Tritordeum (HT621 Advanced Line)	
		*k* (×10^−3^ Month^−1^)	*R*^2^	*k* (×10^−3^ Month^−1^)	*R*^2^
Lutein monolinoleate	20	-	-	-	-
	37	210	0.96	113	0.87
	50	262	0.96	388	0.90
Lutein-3′-*O*-linoleate	20	-	-	-	-
	37	184	0.94	103	0.91
	50	230	0.90	487	0.98
Lutein-3-*O*-linoleate	20	-	-	22	0.92
	37	200	0.95	140	0.94
	50	284	0.91	526	0.96
Lutein monopalmitate	20	-	-	34	0.97
	37	204	0.95	158	0.98
	50	332	0.94	544	0.96
Lutein-3′*-O*-palmitate	20	-	-	-	-
	37	201	0.95	147	0.97
	50	329	0.87	606	0.98
Lutein-3*-O*-palmitate	20	-	-	59	0.98
	37	186	0.96	228	1
	50	336	0.96	632	0.96
Total monoesters	20	-	-	13	0.78
	37	208	0.95	114	0.91
	50	310	0.94	471	0.95
Lutein-3,3′-dilinoleate	20	-	-	-	-
	37	111	0.90	85	0.92
	50	188	0.97	370	0.97
Lutein-3′-*O*-linoleate-3-*O*-palmitate plus Lutein-3′-*O*-palmitate-3-*O*-linoleate	20	-	-	-	-
	37	139	0.95	103	0.96
	50	201	0.96	400	0.97
Lutein-3,3′-dipalmitate	20	-	-	-	-
	37	90	0.78	66	0.83
	50	156	0.96	451	0.98
Total diesters	20	-	-	-	-
	37	122	0.93	88	0.93
	50	190	0.98	395	0.97
Total esters	20	-	-	-	-
	37	162	0.96	-	-
	50	333	0.81	472	0.98

## Supplementary Materials

The following are available online at http://www.mdpi.com/2304-8158/6/12/111/s1, Figure S1: HPLC chromatograms obtained during long-term storage (12 months) at five different temperatures (−32, 6, 20, 37 and 50 °C) of durum wheat (Don Pedro) and tritordeum (HT621) whole-grain flours, Table S1: Evolution of the carotenoid composition (μg/g dry weight) during long-term storage (12 months) of durum wheat (Don Pedro variety) whole-grain flours, Table S2: Evolution of the carotenoid composition (μg/g dry weight) during long-term storage (12 months) of tritordeum (HT621 advanced line) whole-grain flours.
