# Supplementary material for: Lutein Esterification in Wheat Flour Increases the Carotenoid Retention and Is Induced by Storage Temperatures
Source: Foods. 2017 Dec 11;6(12):111. doi: 10.3390/foods6120111 (PMC5742779; doi:10.3390/foods6120111)
Supplement: Supplementary file 1 [file foods-06-00111-s001.zip › Table S2 DEF.pdf]

Table S2. Evolution of the carotenoid composition (μg/g dry weight)[1] during long-term storage (12 months) of tritordeum (HT621 advanced line) whole-grain flours.

Carotenoid	T (°C)	Time (months) 0	1	2	3	4	5	6	7	8	9	10	11	12
(all-*E*)-Lutein	**-32**	3.95 ± 0.04 ab	3.73 ± 0.02 a	3.79 ± 0.01 cd	3.79 ± 0.01 bc	3.84 ± 0.04 e	3.82 ± 0.05 df	3.77 ± 0.03 d	3.73 ± 0.01 g	3.79 ± 0.02 df	3.76 ± 0.01 cd	3.88 ± 0.01 f	3.78 ± 0.01 d	3.69 ± 0.02 df
	**6**	ab	3.55 ± 0.02 a	3.65 ± 0.01 c	3.65 ± 0.03 b	3.62 ± 0.01 ab	3.54 ± 0.02 a	3.47 ± 0.02 d	3.57 ± 0.02 c	3.54 ± 0.01 ab	3.42 ± 0.02 de	3.45 ± 0.02 d	3.36 ± 0.02 e	3.13 ± 0.01 f
	**20**	a	3.42 ± 0.02 b	3.22 ± 0.02 c	2.98 ± 0.02 d	2.70 ± 0.01 e	2.49 ± 0.02 f	2.21 ± 0.05 g	2.13 ± 0.00 h	1.86 ± 0.01 i	1.71 ± 0.01 j	1.68 ± 0.01 j	1.52 ± 0.01 k	1.51 ± 0.01 k
	**37**	a	2.11 ± 0.02 b	1.40 ± 0.01 c	0.93 ± 0.01 d	0.67 ± 0.02 e	0.48 ± 0.02 f	0.36 ± 0.01 g	0.26 ± 0.00 h	0.19 ± 0.00 i	0.13 ± 0.00 j	0.11 ± 0.00 j	0.07 ± 0.00 j	0.07 ± 0.00 j
	**50**	a	0.76 ± 0.01 b	0.26 ± 0.01 c	0.17 ± 0.00 d	0.04 ± 0.00 e	0.05 ± 0.00 e	0.05 ± 0.00 e	0.02 ± 0.00 e	0.01 ± 0.00 e	0.01 ± 0.00 e	-	-	-
Total (*Z*)-Lutein	**-32**	0.49 ± 0.01 abc	0.45 ± 0.00 d	0.44 ± 0.01 ab	0.44 ± 0.00 a	0.42 ± 0.01 d	0.41 ± 0.00 e	0.42 ± 0.00 abcf	0.44 ± 0.01 g	0.43 ± 0.00 cf	0.43 ± 0.00 abcf	0.44 ± 0.02 f	0.43 ± 0.00 cf	0.43 ± 0.00 bcf
	**6**	ab	0.44 ± 0.01 c	0.44 ± 0.00 ade	0.44 ± 0.00 ade	0.42 ± 0.00 c	0.42 ± 0.01 c	0.42 ± 0.00 cd	0.43 ± 0.00 b	0.44 ± 0.00 a	0.42 ± 0.01 cd	0.44 ± 0.00 ae	0.43 ± 0.00 cde	0.41 ± 0.00 c
	**20**	a	0.46 ± 0.00 a	0.44 ± 0.00 a	0.43 ± 0.00 a	0.40 ± 0.01 bc	0.40 ± 0.00 b	0.38 ± 0.00 d	0.37 ± 0.00 cd	0.36 ± 0.00 d	0.33 ± 0.00 e	0.32 ± 0.00 ef	0.31 ± 0.01 f	0.32 ± 0.00 ef
	**37**	a	0.50 ± 0.00 b	0.39 ± 0.01 c	0.26 ± 0.01 d	0.20 ± 0.00 e	0.16 ± 0.01 f	0.12 ± 0.00 g	0.09 ± 0.00 h	0.06 ± 0.00 i	0.05 ± 0.01 j	0.03 ± 0.00 jk	0.03 ± 0.00 k	0.02 ± 0.01 k
	**50**	a	0.27 ± 0.01 b	0.12 ± 0.01 c	0.08 ± 0.00 d	0.04 ± 0.01 e	0.03 ± 0.00 f	0.02 ± 0.01 f	0.00 ± 0.00 e	0.00 ± 0.01 e	0.00 ± 0.00 e	-	-	-
Lutein monolinoleate	**-32**	0.87 ± 0.00 a	0.84 ± 0.00 a	0.86 ± 0.01 bcde	0.84 ± 0.00 bc	0.83 ± 0.00 a	0.83 ± 0.01 b	0.87 ± 0.00 cde	0.89 ± 0.00 f	0.88 ± 0.01 de	0.85 ± 0.02 bcd	0.89 ± 0.03 e	0.85 ± 0.00 bc	0.85 ± 0.00 bc
	**6**		0.86 ± 0.01 a	0.90 ± 0.00 bc	0.87 ± 0.00 b	0.83 ± 0.00 d	0.88 ± 0.01 b	0.90 ± 0.02 bc	0.92 ± 0.01 e	0.92 ± 0.00 e	0.92 ± 0.01 cfg	0.93 ± 0.00 f	0.93 ± 0.00 fg	0.91 ± 0.00 c
	**20**		0.91 ± 0.00 b	0.97 ± 0.01 cd	0.99 ± 0.00 c	0.95 ± 0.01 d	1.00 ± 0.01 c	1.00 ± 0.01 c	1.05 ± 0.00 e	1.05 ± 0.01 f	1.03 ± 0.01 g	1.04 ± 0.00 fg	0.98 ± 0.00 c	0.94 ± 0.00 d
	**37**		1.15 ± 0.01 b	1.16 ± 0.01 b	1.03 ± 0.01 c	0.92 ± 0.01 d	0.84 ± 0.01 e	0.79 ± 0.00 a	0.70 ± 0.00 f	0.59 ± 0.02 g	0.47 ± 0.01 h	0.41 ± 0.00 i	0.30 ± 0.00 j	0.30 ± 0.02 j
	**50**		1.04 ± 0.00 b	0.66 ± 0.01 c	0.41 ± 0.00 d	0.19 ± 0.00 e	0.13 ± 0.01 e	0.09 ± 0.00 e	0.03 ± 0.01 f	0.00 ± 0.00 g	0.00 ± 0.00 g	-	-	-
Lutein 3'-*O*-linoleate	**-32**	0.15 ± 0.00 a	0.14 ± 0.00 a	0.15 ± 0.00 bc	0.15 ± 0.00 b	0.14 ± 0.00 a	0.13 ± 0.00 a	0.15 ± 0.00 bc	0.16 ± 0.00 d	0.16 ± 0.00 cd	0.15 ± 0.00 b	0.16 ± 0.00 cd	0.15 ± 0.00 b	0.15 ± 0.00 b
	**6**		0.14 ± 0.00 a	0.18 ± 0.00 b	0.18 ± 0.00 b	0.16 ± 0.00 c	0.18 ± 0.00 b	0.20 ± 0.00 d	0.22 ± 0.00 e	0.23 ± 0.00 f	0.22 ± 0.00 e	0.23 ± 0.00 f	0.23 ± 0.00 f	0.25 ± 0.00 g
	**20**		0.20 ± 0.00	0.24 ± 0.00	0.27 ± 0.00	0.27 ± 0.00	0.31 ± 0.00	0.34 ± 0.00	0.40 ± 0.00	0.40 ± 0.00	0.42 ± 0.00	0.42 ± 0.00	0.41 ± 0.00	0.39 ± 0.00

			b	c	d	d	e	f	g	h	g	i	h	j
	**37**		0.42 ± 0.00	0.49 ± 0.00	0.47 ± 0.00	0.43 ± 0.00	0.42 ± 0.01	0.42 ± 0.00	0.39 ± 0.00	0.33 ± 0.01	0.27 ± 0.00	0.24 ± 0.00	0.19 ± 0.00	0.19 ± 0.00
			b	c	d	b	b	b	e	f	g	h	i	i
	**50**		0.47 ± 0.00	0.34 ± 0.00	0.20 ± 0.00	0.10 ± 0.00	0.07 ± 0.00	0.06 ± 0.00	0.02 ± 0.00	0.00 ± 0.00	0.00 ± 0.00	-	-	
			b	c	d	e	f	f	g	h	h			
Lutein 3-*O*-linoleate	**-32**	0.72 ± 0.01	0.70 ± 0.01	0.71 ± 0.00	0.69 ± 0.01	0.69 ± 0.01	0.70 ± 0.01	0.72 ± 0.01	0.73 ± 0.00	0.73 ± 0.01	0.70 ± 0.00	0.73 ± 0.00	0.69 ± 0.00	0.70 ± 0.00
		a	a	bcd	b	a	b	bcd	e	cd	bc	d	b	bc
	**6**		0.72 ± 0.00	0.72 ± 0.00	0.70 ± 0.01	0.67 ± 0.00	0.70 ± 0.00	0.70 ± 0.00	0.70 ± 0.00	0.69 ± 0.00	0.70 ± 0.00	0.70 ± 0.00	0.70 ± 0.00	0.65 ± 0.00
		ab	a	c	d	b	d	d	e	e	d	cd	d	ab
	**20**		0.71 ± 0.01	0.72 ± 0.01	0.72 ± 0.00	0.68 ± 0.00	0.68 ± 0.00	0.66 ± 0.01	0.66 ± 0.00	0.65 ± 0.00	0.61 ± 0.01	0.61 ± 0.00	0.58 ± 0.00	0.55 ± 0.00
		ab	c	c	c	d	d	a	c	bd	e	e	f	g
	**37**		0.74 ± 0.00	0.67 ± 0.00	0.56 ± 0.00	0.49 ± 0.00	0.41 ± 0.02	0.37 ± 0.00	0.31 ± 0.00	0.25 ± 0.00	0.19 ± 0.00	0.17 ± 0.00	0.12 ± 0.00	0.12 ± 0.00
		a	b	a	c	d	e	f	g	h	i	j	k	k
	**50**		0.57 ± 0.00	0.32 ± 0.00	0.22 ± 0.00	0.09 ± 0.00	0.06 ± 0.00	0.04 ± 0.00	0.01 ± 0.00	0.00 ± 0.00	0.00 ± 0.00	-	-	-
		a	b	c	d	e	e	e	f	f	f			
Lutein	**-32**	1.50 ± 0.01	1.46 ± 0.01	1.49 ± 0.01	1.46 ± 0.00	1.44 ± 0.00	1.44 ± 0.02	1.50 ± 0.00	1.50 ± 0.01	1.52 ± 0.01	1.47 ± 0.03	1.50 ± 0.05	1.45 ± 0.00	1.44 ± 0.00
monopalmitate		ab	a	cd	ef	a	bg	cde	h	cd	cef	d	efg	fg
	**6**		1.45 ± 0.03	1.49 ± 0.00	1.42 ± 0.00	1.36 ± 0.01	1.39 ± 0.02	1.40 ± 0.03	1.45 ± 0.02	1.43 ± 0.00	1.42 ± 0.01	1.42 ± 0.00	1.38 ± 0.00	1.30 ± 0.00
		ab	a	c	d	a	be	bde	c	c	de	de	be	f
	**20**		1.45 ± 0.00	1.45 ± 0.01	1.39 ± 0.00	1.27 ± 0.02	1.25 ± 0.01	1.18 ± 0.01	1.24 ± 0.01	1.18 ± 0.01	1.09 ± 0.00	1.06 ± 0.00	1.03 ± 0.01	0.99 ± 0.00
		a	b	b	a	cd	ce	f	e	df	g	g	h	i
	**37**		1.40 ± 0.01	1.26 ± 0.01	0.99 ± 0.02	0.80 ± 0.01	0.71 ± 0.01	0.66 ± 0.01	0.57 ± 0.01	0.46 ± 0.03	0.36 ± 0.01	0.29 ± 0.00	0.22 ± 0.01	0.21 ± 0.02
		a	a	b	c	d	e	f	g	h	i	j	k	k
	**50**		1.10 ± 0.01	0.60 ± 0.02	0.39 ± 0.01	0.16 ± 0.00	0.10 ± 0.01	0.08 ± 0.00	0.02 ± 0.01	0.00 ± 0.00	0.00 ± 0.01	-	-	-
		a	b	c	d	e	e	e	f	f	f			
Luteín 3’-*O*-	**-32**	0.49 ± 0.01	0.47 ± 0.01	0.49 ± 0.00	0.49 ± 0.01	0.46 ± 0.01	0.48 ± 0.01	0.50 ± 0.00	0.49 ± 0.00	0.51 ± 0.00	0.50 ± 0.00	0.52 ± 0.00	0.49 ± 0.00	0.48 ± 0.00
palmitate		a	a	bc	bde	a	a	bd	f	bc	bde	c	de	e
	**6**		0.48 ± 0.00	0.50 ± 0.00	0.48 ± 0.01	0.47 ± 0.00	0.47 ± 0.00	0.50 ± 0.00	0.51 ± 0.00	0.51 ± 0.00	0.52 ± 0.00	0.52 ± 0.00	0.51 ± 0.00	0.47 ± 0.00
			a	b	c	a	d	c	e	e	b	b	bc	d
	**20**		0.51 ± 0.01	0.54 ± 0.00	0.53 ± 0.00	0.50 ± 0.00	0.50 ± 0.00	0.50 ± 0.01	0.52 ± 0.00	0.52 ± 0.00	0.52 ± 0.01	0.51 ± 0.00	0.51 ± 0.00	0.50 ± 0.00
			b	c	c	d	b	b	e	c	b	b	d	a
	**37**		0.61 ± 0.00	0.61 ± 0.00	0.52 ± 0.00	0.43 ± 0.00	0.40 ± 0.01	0.39 ± 0.00	0.35 ± 0.00	0.29 ± 0.00	0.23 ± 0.00	0.19 ± 0.00	0.15 ± 0.00	0.14 ± 0.00
			b	b	c	d	e	e	f	g	h	i	j	j
	**50**		0.57 ± 0.00	0.35 ± 0.00	0.22 ± 0.00	0.09 ± 0.00	0.06 ± 0.00	0.04 ± 0.00	0.01 ± 0.00	0.00 ± 0.00	0.00 ± 0.00	-	-	-
			b	c	d	e	e	e	f	f	f			
Lutein 3-*O*-palmitate	**-32**	1.01 ± 0.02	0.99 ± 0.01	1.00 ± 0.01	0.96 ± 0.01	0.97 ± 0.01	0.96 ± 0.02	1.00 ± 0.01	1.01 ± 0.00	1.01 ± 0.01	0.98 ± 0.00	0.98 ± 0.00	0.96 ± 0.01	0.96 ± 0.00
		a	a	bc	de	a	d	bce	f	bc	bde	c	d	d
	**6**		0.97 ± 0.01	0.99 ± 0.00	0.94 ± 0.01	0.89 ± 0.00	0.92 ± 0.01	0.90 ± 0.00	0.94 ± 0.00	0.93 ± 0.00	0.90 ± 0.01	0.90 ± 0.00	0.87 ± 0.00	0.83 ± 0.00
		abc	ad	e	cf	ad	bc	ab	eg	fg	ab	ab	d	h
	**20**		0.94 ± 0.01	0.92 ± 0.00	0.85 ± 0.00	0.77 ± 0.01	0.75 ± 0.01	0.68 ± 0.01	0.72 ± 0.00	0.66 ± 0.01	0.57 ± 0.01	0.55 ± 0.00	0.52 ± 0.00	0.49 ± 0.00
		a	a	a	b	c	c	d	e	d	f	f	g	h
	**37**		0.79 ± 0.00	0.65 ± 0.00	0.47 ± 0.00	0.37 ± 0.00	0.30 ± 0.01	0.26 ± 0.00	0.21 ± 0.00	0.17 ± 0.00	0.13 ± 0.00	0.10 ± 0.00	0.08 ± 0.00	0.07 ± 0.00
		a	b	c	d	e	f	g	h	i	j	k	l	l

	**50**	a	0.52 ± 0.00 b	0.25 ± 0.00 c	0.18 ± 0.00 d	0.07 ± 0.00 e	0.04 ± 0.00 e	0.04 ± 0.00 e	0.01 ± 0.00 f	0.00 ± 0.00 f	0.00 ± 0.00 f	-	-	-
(all-*E*)-β-Carotene	**-32**	0.06 ± 0.00 a	0.06 ± 0.00 a	0.06 ± 0.00 bcd	0.06 ± 0.00 bcd	0.07 ± 0.00 a	0.07 ± 0.00 e	0.07 ± 0.00 bce	0.07 ± 0.00 be	0.06 ± 0.00 bcd	0.06 ± 0.00 bcd	0.06 ± 0.00 bce	0.06 ± 0.00 ad	0.06 ± 0.00 acd
	**6**	ab	0.06 ± 0.00 abc	0.06 ± 0.00 d	0.06 ± 0.00 def	0.06 ± 0.00 def	0.06 ± 0.00 cef	0.06 ± 0.00 ef	0.06 ± 0.00 de	0.06 ± 0.00 de	0.06 ± 0.00 def	0.06 ± 0.00 def	0.06 ± 0.00 acf	0.06 ± 0.00 b
	**20**	ab	0.06 ± 0.00 c	0.06 ± 0.00 c	0.06 ± 0.00 ac	0.05 ± 0.00 bd	0.05 ± 0.00 bde	0.05 ± 0.00 def	0.05 ± 0.00 bd	0.05 ± 0.00 bde	0.05 ± 0.00 efg	0.05 ± 0.00 fg	0.04 ± 0.00 gh	0.04 ± 0.00 h
	**37**	a	0.06 ± 0.00 a	0.05 ± 0.00 b	0.04 ± 0.00 c	0.04 ± 0.00 c	0.03 ± 0.00 d	0.03 ± 0.00 d	0.03 ± 0.00 d	0.02 ± 0.00 e	0.02 ± 0.00 ef	0.02 ± 0.00 f	0.01 ± 0.00 g	0.01 ± 0.00 g
	**50**	a	0.04 ± 0.00 b	0.03 ± 0.00 c	0.02 ± 0.00 d	0.00 ± 0.00 e	0.00 ± 0.00 e	0.00 ± 0.00 e	0.00 ± 0.00 e	0.00 ± 0.00 e	0.00 ± 0.00 e	-	-	-
Lutein dilinoleate	**-32**	0.12 ± 0.00 abcde	0.11 ± 0.00 abc	0.12 ± 0.01 de	0.12 ± 0.00 ade	0.10 ± 0.00 bc	0.11 ± 0.01 abcd	0.13 ± 0.01 e	0.14 ± 0.01 f	0.13 ± 0.00 e	0.11 ± 0.01 abcd	0.12 ± 0.00 abde	0.11 ± 0.00 abc	0.10 ± 0.01 c
	**6**		0.12 ± 0.01 a	0.14 ± 0.00 b	0.15 ± 0.01 b	0.14 ± 0.00 b	0.18 ± 0.00 c	0.19 ± 0.00 c	0.23 ± 0.00 de	0.23 ± 0.01 d	0.22 ± 0.00 d	0.22 ± 0.00 d	0.22 ± 0.00 d	0.24 ± 0.00 e
	**20**		0.16 ± 0.00 b	0.20 ± 0.01 c	0.26 ± 0.01 d	0.28 ± 0.01 d	0.35 ± 0.01 e	0.39 ± 0.01 f	0.51 ± 0.00 gh	0.50 ± 0.01 g	0.53 ± 0.01 h	0.55 ± 0.00 i	0.50 ± 0.00 g	0.53 ± 0.01 h
	**37**		0.42 ± 0.01 b	0.55 ± 0.01 c	0.67 ± 0.00 d	0.74 ± 0.02 e	0.79 ± 0.02 f	0.96 ± 0.01 g	1.02 ± 0.01 h	0.93 ± 0.02 i	0.82 ± 0.01 f	0.82 ± 0.01 j	0.67 ± 0.00 d	0.69 ± 0.01 d
	**50**		0.69 ± 0.03 b	0.76 ± 0.01 c	0.54 ± 0.01 d	0.37 ± 0.00 e	0.30 ± 0.00 ef	0.23 ± 0.01 f	0.12 ± 0.00 a	0.07 ± 0.00 g	0.04 ± 0.00 g	-	-	-
Lutein 3’-*O*-linoleate-3-*O*-palmitate and Lutein 3’-*O*-palmitate-3-*O*-linoleate	**-32**	0.42 ± 0.01 abcd	0.38 ± 0.01 abcd	0.41 ± 0.00 cef	0.39 ± 0.00 abcde	0.36 ± 0.00 a	0.38 ± 0.01 ab	0.42 ± 0.01 bcdef	0.43 ± 0.00 ef	0.42 ± 0.01 f	0.39 ± 0.00 abcd	0.43 ± 0.01 cdef	0.38 ± 0.01 abd	0.37 ± 0.01 ab
	**6**	a	0.38 ± 0.01 a	0.45 ± 0.01 b	0.42 ± 0.00 b	0.42 ± 0.00 b	0.48 ± 0.01 c	0.48 ± 0.00 cd	0.54 ± 0.00 e	0.54 ± 0.01 e	0.51 ± 0.00 fg	0.51 ± 0.00 ef	0.49 ± 0.00 dfg	0.47 ± 0.01 cdg
	**20**	a	0.47 ± 0.01 b	0.51 ± 0.00 c	0.55 ± 0.00 d	0.54 ± 0.00 d	0.63 ± 0.01 e	0.63 ± 0.00 e	0.76 ± 0.01 fg	0.75 ± 0.02 f	0.72 ± 0.01 fh	0.73 ± 0.01 g	0.67 ± 0.00 i	0.69 ± 0.01 hi
	**37**	a	0.73 ± 0.01 b	0.84 ± 0.01 c	0.90 ± 0.01 d	0.93 ± 0.03 de	0.97 ± 0.03 f	1.08 ± 0.01 g	1.10 ± 0.02 h	1.00 ± 0.02 i	0.86 ± 0.00 ef	0.81 ± 0.01 ef	0.68 ± 0.00 c	0.69 ± 0.01 c
	**50**	a	1.06 ± 0.01 b	0.97 ± 0.01 c	0.72 ± 0.00 d	0.42 ± 0.00 e	0.34 ± 0.00 e	0.26 ± 0.01 e	0.14 ± 0.00 f	0.08 ± 0.00 g	0.04 ± 0.00 g	-	-	-
Lutein dipalmitate	-32	0.41 ± 0.01 abc	0.36 ± 0.00 ad	0.39 ± 0.00 bc	0.37 ± 0.01 ab	0.34 ± 0.01 ad	0.36 ± 0.01 ad	0.39 ± 0.01 bc	0.43 ± 0.01 e	0.40 ± 0.01 c	0.39 ± 0.00 bc	0.40 ± 0.01 c	0.36 ± 0.00 ad	0.34 ± 0.00 d
	**6**	a	0.34 ± 0.00 b	0.39 ± 0.00 ac	0.37 ± 0.00 a	0.34 ± 0.00 b	0.39 ± 0.01 acd	0.39 ± 0.00 acd	0.43 ± 0.01 e	0.41 ± 0.01 ef	0.40 ± 0.01 df	0.39 ± 0.01 cdf	0.36 ± 0.00 a	0.31 ± 0.00 b
	**20**	a	0.38 ± 0.01 ab	0.39 ± 0.00 abc	0.39 ± 0.00 bc	0.35 ± 0.00 a	0.39 ± 0.01 cd	0.36 ± 0.01 abc	0.42 ± 0.01 e	0.39 ± 0.01 de	0.37 ± 0.00 d	0.36 ± 0.00 d	0.32 ± 0.01 ab	0.32 ± 0.01 ab
	**37**	a	0.44 ± 0.01 bc	0.44 ± 0.00 bd	0.41 ± 0.01 bd	0.37 ± 0.01 bc	0.37 ± 0.02 bc	0.39 ± 0.01 d	0.37 ± 0.01 d	0.33 ± 0.01 bd	0.28 ± 0.00 c	0.25 ± 0.00 e	0.21 ± 0.00 f	0.19 ± 0.00 f
	**50**	a	0.51 ± 0.00	0.39 ± 0.01	0.30 ± 0.00	0.16 ± 0.00	0.12 ± 0.00	0.08 ± 0.00	0.04 ± 0.00	0.03 ± 0.00	0.01 ± 0.00	-	-	-

			b	c	d	e	e	e	f	g	fg			
**Monoesters and Regioisomers ratios**														
Lutein monopalmitate/ Lutein monolinoleate	**-32**	1.72	1.74	1.73	1.73	1.74	1.73	1.72	1.68	1.72	1.73	1.69	1.71	1.69
	**6**		1.69	1.66	1.62	1.62	1.58	1.56	1.58	1.56	1.54	1.52	1.48	1.44
	**20**		1.58	1.50	1.40	1.34	1.26	1.19	1.17	1.13	1.06	1.02	1.05	1.05
	**37**		1.21	1.08	0.96	0.88	0.84	0.83	0.81	0.79	0.77	0.72	0.74	0.69
	**50**		1.05	0.91	0.95	0.85	0.74	0.89	0.60	-	-	-	-	-
Lutein 3-*O*-linoleate /Lutein 3’-*O*-linoleate	**-32**	4.68	5.15	4.59	4.58	5.01	5.20	4.70	4.44	4.57	4.68	4.58	4.58	4.69
	**6**		5.03	4.11	3.96	4.11	3.91	3.49	3.24	3.07	3.22	3.05	3.01	2.59
	**20**		3.56	2.97	2.63	2.52	2.19	1.93	1.65	1.63	1.45	1.44	1.43	1.43
	**37**		1.77	1.39	1.20	1.15	0.98	0.90	0.82	0.76	0.70	0.70	0.64	0.64
	**50**		1.20	0.92	1.12	0.86	0.77	0.68	0.47	-	-	-	-	-
Lutein 3-*O*-palmitate /Lutein 3’-*O*-palmitate	**-32**	2.05	2.11	2.04	1.96	2.11	2.01	2.00	2.04	1.99	1.98	1.89	1.97	2.00
	**6**		2.01	1.99	1.95	1.90	1.96	1.82	1.83	1.82	1.74	1.72	1.70	1.78
	**20**		1.83	1.70	1.60	1.52	1.48	1.34	1.39	1.27	1.11	1.08	1.02	0.97
	**37**		1.30	1.07	0.92	0.87	0.75	0.66	0.61	0.59	0.55	0.53	0.52	0.51
	**50**		0.92	0.72	0.81	0.78	0.74	0.99	0.61	-	-	-	-	-

[1]Data are the mean ± standard error (n=5 for the starting sample, n=3 for the rest of the samples). Different letters within the same line (for each pigment) indicate significant differences ($p<0.05$) determined by the Duncan test.
