# Supplementary material for: Lutein Esterification in Wheat Flour Increases the Carotenoid Retention and Is Induced by Storage Temperatures
Source: Foods. 2017 Dec 11;6(12):111. doi: 10.3390/foods6120111 (PMC5742779; doi:10.3390/foods6120111)
Supplement: Supplementary file 1 [file foods-06-00111-s001.zip › Figure S1 colour DEF Foods.pdf]

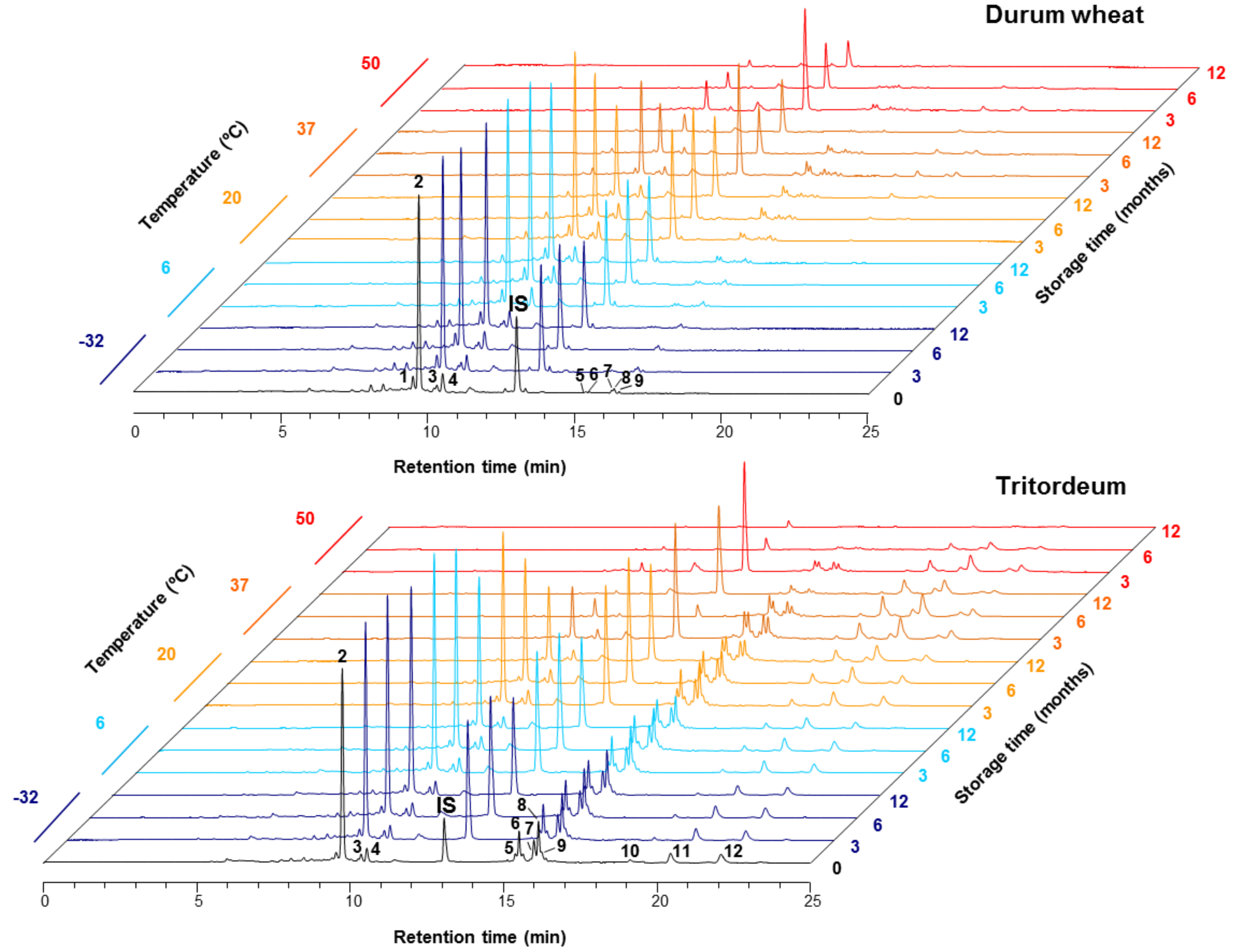

Supplementary Figure 1 (Figure S1). HPLC chromatograms obtained during long-term storage (12 months) at five different temperatures (-32, 6, 20, 37 and 50 °C) of durum wheat (Don Pedro) and tritordeum (HT621) whole-grain flours. Peak identities: 1, (all-*E*)-Zeaxanthin; 2, (all-*E*)-Lutein; 3, (9*Z*)-Lutein; 4, (13*Z*)-Lutein; 5, Lutein 3'-*O*-linoleate; 6, Lutein 3-*O*-linoleate; 7, Lutein 3'-*O*-palmitate; 8, Lutein 3-*O*-palmitate; 9, (all-E)-β-Carotene; 10, Lutein dilinoleate; 11, Lutein 3'-*O*-linoleate-3-*O*-palmitate and Lutein 3'-*O*-palmitate-3-*O*-linoleate; 12, Lutein dipalmitate. Internal Standard (IS): β-Apo-8'-carotenal.
