# Supplementary material for: Lutein Esterification in Wheat Flour Increases the Carotenoid Retention and Is Induced by Storage Temperatures
Source: Foods. 2017 Dec 11;6(12):111. doi: 10.3390/foods6120111 (PMC5742779; doi:10.3390/foods6120111)
Supplement: Supplementary file 1 [file foods-06-00111-s001.zip › Table S1 DEF.pdf]

Table S1. Evolution of the carotenoid composition (μg/g dry weight)[1] during long-term storage (12 months) of durum wheat (Don Pedro variety) whole-grain flours.

Carotenoid	T (°C)	Initial content (μg/g)	Time (months) 1	2	3	4	5	6	7	8	9	10	11	12
(All-*E*)-Zeaxanthin	**-32**	0.08 ± 0.00 a	0.08 ± 0.00 ab	0.07 ± 0.00 ab	0.08 ± 0.00 a	0.08 ± 0.00 a	0.07 ± 0.00 ab	0.07 ± 0.00 ab	0.08 ± 0.00 b	0.08 ± 0.00 b	0.08 ± 0.00 b	0.08 ± 0.00 ab	0.08 ± 0.00 ab	0.08 ± 0.00 b
	**6**	a	0.08 ± 0.00 abc	0.08 ± 0.00 d	0.07 ± 0.00 bc	0.07 ± 0.00 abc	0.07 ± 0.00 ab	0.07 ± 0.00 abc	0.07 ± 0.00 d	0.07 ± 0.00 abc	0.07 ± 0.00 c	0.07 ± 0.00 bc	0.07 ± 0.00 a	0.07 ± 0.00 a
	**20**	ab	0.07 ± 0.00 c	0.07 ± 0.00 a	0.07 ± 0.01 b	0.06 ± 0.00 d	0.06 ± 0.00 d	0.05 ± 0.00 e	0.05 ± 0.00 d	0.04 ± 0.00 e	0.04 ± 0.00 e	0.04 ± 0.00 e	0.03 ± 0.00 f	0.03 ± 0.00 f
	**37**	a	0.06 ± 0.00 b	0.03 ± 0.00 c	0.03 ± 0.00 d	0.02 ± 0.00 e	0.02 ± 0.00 f	0.01 ± 0.00 e	0.01 ± 0.00 e	0.01 ± 0.00 g	0.01 ± 0.00 g	0.01 ± 0.00 g	0.00 ± 0.00 h	0.00 ± 0.00 h
	**50**	a	0.02 ± 0.00 b	0.01 ± 0.00 c	0.01 ± 0.00 cd	0.00 ± 0.00 de	0.00 ± 0.00 f	0.00 ± 0.00 ef	0.00 ± 0.00 f	0.00 ± 0.00 f	0.00 ± 0.00 f	-	-	-
(all-*E*)-Lutein	**-32**	1.08 ± 0.02 abcd	1.06 ± 0.01 ae	1.04 ± 0.02 abe	1.05 ± 0.02 abce	1.05 ± 0.01 e	1.06 ± 0.02 abcd	1.05 ± 0.01 bcdf	1.09 ± 0.03 f	1.09 ± 0.04 f	1.05 ± 0.02 bcdf	1.07 ± 0.00 cdf	1.07 ± 0.00 df	1.08 ± 0.02 df
	**6**	abc	0.98 ± 0.01 a	1.02 ± 0.01 def	1.03 ± 0.00 abc	1.03 ± 0.01 abc	1.04 ± 0.05 de	1.04 ± 0.01 bcf	1.04 ± 0.02 d	1.04 ± 0.01 ef	0.98 ± 0.01 bcef	1.03 ± 0.02 bef	1.00 ± 0.01 abc	0.98 ± 0.01 ac
	**20**	a	0.99 ± 0.00 b	0.95 ± 0.01 a	0.92 ± 0.02 c	0.86 ± 0.01 d	0.85 ± 0.01 d	0.79 ± 0.00 e	0.79 ± 0.00 d	0.73 ± 0.01 e	0.71 ± 0.01 e	0.67 ± 0.00 f	0.56 ± 0.01 g	0.53 ± 0.00 g
	**37**	a	0.77 ± 0.01 b	0.60 ± 0.01 c	0.47 ± 0.01 d	0.32 ± 0.00 e	0.32 ± 0.01 f	0.27 ± 0.01 g	0.23 ± 0.01 h	0.17 ± 0.00 i	0.15 ± 0.00 i	0.16 ± 0.00 i	0.10 ± 0.00 j	0.10 ± 0.00 j
	**50**	a	0.30 ± 0.00 b	0.17 ± 0.00 c	0.16 ± 0.00 cd	0.13 ± 0.00 d	0.07 ± 0.00 e	0.09 ± 0.00 e	0.06 ± 0.00 ef	0.07 ± 0.00 e	0.03 ± 0.00 f	-	-	-
Total (*Z*)-Lutein	**-32**	0.18 ± 0.00 abc	0.17 ± 0.00 d	0.17 ± 0.00 ab	0.16 ± 0.00 ad	0.16 ± 0.00 abc	0.16 ± 0.00 ab	0.17 ± 0.00 bc	0.17 ± 0.00 e	0.18 ± 0.00 f	0.17 ± 0.00 ab	0.17 ± 0.00 f	0.17 ± 0.00 c	0.16 ± 0.00 abc
	**6**	abc	0.16 ± 0.00 abcd	0.15 ± 0.00 d	0.17 ± 0.00 abc	0.16 ± 0.00 acd	0.15 ± 0.01 e	0.16 ± 0.00 abcd	0.16 ± 0.01 b	0.17 ± 0.00 ab	0.16 ± 0.00 cd	0.17 ± 0.00 abc	0.16 ± 0.00 abc	0.16 ± 0.00 abcd
	**20**	a	0.15 ± 0.00 b	0.15 ± 0.00 b	0.16 ± 0.00 b	0.14 ± 0.00 c	0.14 ± 0.00 b	0.13 ± 0.00 d	0.13 ± 0.00 c	0.12 ± 0.00 d	0.13 ± 0.00 d	0.11 ± 0.00 e	0.11 ± 0.00 e	0.11 ± 0.00 e
	**37**	a	0.16 ± 0.00 a	0.13 ± 0.00 b	0.09 ± 0.00 c	0.07 ± 0.00 d	0.07 ± 0.00 e	0.06 ± 0.00 d	0.05 ± 0.00 f	0.04 ± 0.00 g	0.03 ± 0.00 g	0.04 ± 0.00 g	0.03 ± 0.00 h	0.02 ± 0.00 h
	**50**	a	0.07 ± 0.00 b	0.05 ± 0.00 c	0.05 ± 0.00 d	0.04 ± 0.00 e	0.02 ± 0.00 f	0.03 ± 0.00 g	0.02 ± 0.00 f	0.02 ± 0.00 h	0.01 ± 0.00 i	-	-	-
Lutein monolinoleate	**-32**	0.03 ± 0.00 a	0.02 ± 0.00 a	0.02 ± 0.00 a	0.03 ± 0.00 a	0.03 ± 0.00 a	0.02 ± 0.00 a	0.03 ± 0.00 a	0.03 ± 0.00 b	0.03 ± 0.00 a	0.03 ± 0.00 a	0.03 ± 0.00 a	0.03 ± 0.00 a	0.03 ± 0.00 a

	**6**		0.03 ± 0.00 a	0.03 ± 0.00 b	0.04 ± 0.00 c	0.04 ± 0.00 c	0.05 ± 0.00 d	0.05 ± 0.00 e	0.06 ± 0.00 f	0.06 ± 0.00 g	0.06 ± 0.00 g	0.07 ± 0.00 hi	0.07 ± 0.00 h	0.07 ± 0.00 i
	**20**		0.04 ± 0.01 b	0.06 ± 0.01 c	0.08 ± 0.01 d	0.08 ± 0.00 d	0.09 ± 0.00 e	0.10 ± 0.00 f	0.11 ± 0.00 g	0.11 ± 0.00 g	0.11 ± 0.00 g	0.12 ± 0.00 g	0.10 ± 0.00 f	0.11 ± 0.00 h
	**37**		0.09 ± 0.01 b	0.12 ± 0.01 c	0.12 ± 0.00 c	0.12 ± 0.01 bd	0.11 ± 0.01 c	0.08 ± 0.01 d	0.07 ± 0.00 e	0.05 ± 0.01 f	0.04 ± 0.00 f	0.04 ± 0.00 f	0.03 ± 0.01 a	0.03 ± 0.00 a
	**50**		0.10 ± 0.02 b	0.08 ± 0.01 c	0.06 ± 0.00 d	0.05 ± 0.01 e	0.03 ± 0.01 f	0.03 ± 0.00 g	0.02 ± 0.00 h	0.02 ± 0.00 a	0.01 ± 0.00 i	-	-	-
Lutein 3’-*O*-linoleate	**-32**	0.01 ± 0.00 ab	0.01 ± 0.00 ab	0.01 ± 0.00 ab	0.01 ± 0.00 ab	0.01 ± 0.00 ab	0.01 ± 0.00 ab	0.01 ± 0.00 ab	0.01 ± 0.00 c	0.01 ± 0.00 ab	0.01 ± 0.00 a	0.01 ± 0.00 b	0.01 ± 0.00 ab	0.01 ± 0.00 ab
	**6**	a	0.01 ± 0.00 a	0.02 ± 0.00 b	0.02 ± 0.00 c	0.02 ± 0.00 c	0.02 ± 0.00 d	0.02 ± 0.00 e	0.03 ± 0.00 f	0.03 ± 0.00 g	0.03 ± 0.00 g	0.03 ± 0.00 h	0.03 ± 0.00 h	0.03 ± 0.00 h
	**20**	a	0.02 ± 0.00 b	0.03 ± 0.00 c	0.04 ± 0.00 d	0.04 ± 0.00 d	0.05 ± 0.00 e	0.06 ± 0.00 f	0.06 ± 0.00 g	0.06 ± 0.00 gh	0.06 ± 0.00 gi	0.06 ± 0.00 h	0.06 ± 0.00 i	0.06 ± 0.00 gh
	**37**	a	0.05 ± 0.00 b	0.06 ± 0.00 c	0.06 ± 0.00 c	0.06 ± 0.00 b	0.06 ± 0.00 c	0.05 ± 0.00 b	0.04 ± 0.00 d	0.03 ± 0.00 e	0.03 ± 0.00 e	0.03 ± 0.00 e	0.02 ± 0.00 f	0.02 ± 0.00 af
	**50**	a	0.06 ± 0.00 b	0.04 ± 0.00 c	0.03 ± 0.00 d	0.02 ± 0.00 e	0.01 ± 0.00 a	0.02 ± 0.00 f	0.01 ± 0.00 a	0.01 ± 0.00 a	0.01 ± 0.00 g	-	-	-
Lutein 3-*O*-linoleate	**-32**	0.01 ± 0.00 ab	0.01 ± 0.00 ab	0.01 ± 0.00 a	0.01 ± 0.00 ab	0.01 ± 0.00 a	0.01 ± 0.00 ab	0.01 ± 0.00 ab	0.02 ± 0.00 c	0.01 ± 0.00 ab	0.01 ± 0.00 ab	0.02 ± 0.00 bc	0.01 ± 0.00 ab	0.01 ± 0.00 ab
	**6**	a	0.01 ± 0.00 a	0.02 ± 0.00 b	0.02 ± 0.00 c	0.02 ± 0.00 c	0.02 ± 0.00 d	0.03 ± 0.00 e	0.03 ± 0.00 f	0.03 ± 0.00 g	0.03 ± 0.00 gh	0.04 ± 0.00 ij	0.04 ± 0.00 hi	0.04 ± 0.00 j
	**20**	a	0.02 ± 0.00 b	0.03 ± 0.00 c	0.04 ± 0.00 d	0.04 ± 0.00 e	0.05 ± 0.00 fg	0.05 ± 0.00 f	0.05 ± 0.00 h	0.05 ± 0.00 i	0.05 ± 0.00 hi	0.05 ± 0.00 i	0.04 ± 0.00 g	0.05 ± 0.00 fg
	**37**	ab	0.05 ± 0.00 c	0.06 ± 0.00 d	0.05 ± 0.00 e	0.05 ± 0.00 f	0.05 ± 0.00 e	0.03 ± 0.00 fg	0.03 ± 0.00 g	0.02 ± 0.00 h	0.02 ± 0.00 hi	0.02 ± 0.00 ai	0.01 ± 0.00 j	0.01 ± 0.00 bj
	**50**	a	0.04 ± 0.00 b	0.04 ± 0.00 c	0.03 ± 0.00 c	0.03 ± 0.00 d	0.02 ± 0.00 e	0.02 ± 0.00 f	0.01 ± 0.00 e	0.01 ± 0.00 a	0.00 ± 0.00 g	-	-	-
Lutein monopalmitate	**-32**	0.01 ± 0.00 abc	0.01 ± 0.00 a	0.01 ± 0.00 abc	0.01 ± 0.00 d	0.00 ± 0.00 d	0.01 ± 0.00 d	0.01 ± 0.00 abc	0.01 ± 0.00 bc	0.01 ± 0.00 b	0.01 ± 0.00 ac	0.01 ± 0.00 b	0.01 ± 0.00 abc	0.01 ± 0.00 abc
	**6**	a	0.02 ± 0.00 b	0.02 ± 0.00 c	0.03 ± 0.00 c	0.02 ± 0.00 d	0.03 ± 0.00 c	0.03 ± 0.00 e	0.03 ± 0.00 ef	0.04 ± 0.00 g	0.03 ± 0.00 f	0.03 ± 0.00 f	0.03 ± 0.00 f	0.03 ± 0.00 f
	**20**	a	0.03 ± 0.00 b	0.04 ± 0.00 c	0.05 ± 0.00 d	0.04 ± 0.00 d	0.05 ± 0.00 e	0.06 ± 0.00 f	0.07 ± 0.00 g	0.06 ± 0.00 gh	0.06 ± 0.00 gh	0.06 ± 0.00 h	0.06 ± 0.00 ef	0.06 ± 0.00 ef
	**37**	a	0.06 ± 0.00 b	0.08 ± 0.00 c	0.07 ± 0.00 d	0.07 ± 0.00 e	0.07 ± 0.00 d	0.05 ± 0.00 e	0.04 ± 0.00 e	0.03 ± 0.00 f	0.03 ± 0.00 f	0.03 ± 0.00 f	0.02 ± 0.00 g	0.02 ± 0.00 g
	**50**	a	0.07 ± 0.00 b	0.05 ± 0.00 c	0.04 ± 0.00 d	0.02 ± 0.00 e	0.01 ± 0.00 fg	0.01 ± 0.00 h	0.01 ± 0.00 af	0.01 ± 0.00 g	0.00 ± 0.00 i	-	-	-
Luteín 3’-*O*-palmitate	**-32**	0.00 ± 0.00 abc	0.00 ± 0.00 ab	0.00 ± 0.00 abc	0.00 ± 0.00 d	0.00 ± 0.00 d	0.00 ± 0.00 d	0.00 ± 0.00 abc	0.00 ± 0.00 abc	0.00 ± 0.00 abc	0.00 ± 0.00 a	0.00 ± 0.00 bc	0.00 ± 0.00 c	0.00 ± 0.00 abc
	**6**	a	0.00 ± 0.00	0.01 ± 0.00	0.01 ± 0.00	0.01 ± 0.00	0.01 ± 0.00	0.01 ± 0.00	0.01 ± 0.00	0.01 ± 0.00	0.01 ± 0.00	0.01 ± 0.00	0.01 ± 0.00	0.01 ± 0.00

			a	b	c	bc	d	e	e	f	f	f	f	f
	**20**	a	0.01 ± 0.00 b	0.02 ± 0.00 c	0.02 ± 0.00 d	0.02 ± 0.00 d	0.03 ± 0.00 e	0.03 ± 0.00 f	0.04 ± 0.00 g	0.04 ± 0.00 g	0.04 ± 0.00 g	0.04 ± 0.00 g	0.03 ± 0.00 f	0.03 ± 0.00 f
	**37**	a	0.03 ± 0.00 b	0.04 ± 0.00 c	0.04 ± 0.00 c	0.04 ± 0.00 bd	0.04 ± 0.00 c	0.03 ± 0.00 bd	0.03 ± 0.00 d	0.02 ± 0.00 e	0.02 ± 0.00 e	0.02 ± 0.00 e	0.01 ± 0.00 f	0.01 ± 0.00 f
	**50**	a	0.04 ± 0.00	0.03 ± 0.00	0.02 ± 0.00	0.01 ± 0.00	0.01 ± 0.00	0.01 ± 0.00	0.00 ± 0.00	0.00 ± 0.00	0.00 ± 0.00	0.00 ± 0.00	0.00 ± 0.00	0.00 ± 0.00
Luteín 3-*O*-palmitate	**-32**	0.00 ± 0.00 abcd	0.00 ± 0.00 ae	0.00 ± 0.00 abc	0.00 ± 0.00 ae	0.00 ± 0.00 e	0.00 ± 0.00 e	0.00 ± 0.00 abcd	0.00 ± 0.00 bcd	0.00 ± 0.00 d	0.00 ± 0.00 abe	0.00 ± 0.00 cd	0.00 ± 0.00 abc	0.00 ± 0.00 abcd
	**6**	a	0.02 ± 0.00 b	0.02 ± 0.00 c	0.02 ± 0.00 c	0.02 ± 0.00 b	0.02 ± 0.00 c	0.02 ± 0.00 d	0.02 ± 0.00 d	0.02 ± 0.00 e	0.02 ± 0.00 d	0.02 ± 0.00 d	0.02 ± 0.00 d	0.02 ± 0.00 d
	**20**	a	0.02 ± 0.00 b	0.02 ± 0.00 cde	0.02 ± 0.00 cdf	0.02 ± 0.00 ce	0.03 ± 0.00 fgh	0.02 ± 0.00 fgh	0.03 ± 0.00 i	0.03 ± 0.00 gh	0.03 ± 0.00 g	0.02 ± 0.00 dfh	0.02 ± 0.00 cde	0.02 ± 0.00 e
	**37**	a	0.03 ± 0.00 b	0.04 ± 0.00 c	0.03 ± 0.00 d	0.03 ± 0.00 e	0.03 ± 0.00 bd	0.02 ± 0.00 e	0.02 ± 0.00 e	0.01 ± 0.00 f	0.01 ± 0.00 f	0.01 ± 0.00 h	0.01 ± 0.00 g	0.01 ± 0.00 ag
	**50**	a	0.03 ± 0.00 b	0.02 ± 0.00 c	0.02 ± 0.00 d	0.01 ± 0.00 e	0.01 ± 0.00 a	0.00 ± 0.00 f	0.00 ± 0.00 a	0.00 ± 0.00 g	0.00 ± 0.00 h	-	-	-
(all-*E*)-β-Carotene	**-32**	0.02 ± 0.00 ab	0.03 ± 0.00 a	0.03 ± 0.00 cde	0.03 ± 0.00 abc	0.02 ± 0.00 ab	0.02 ± 0.00 abcd	0.03 ± 0.00 e	0.03 ± 0.00 f	0.03 ± 0.00 de	0.03 ± 0.00 abc	0.03 ± 0.00 e	0.03 ± 0.00 de	0.02 ± 0.00 bcde
	**6**	a	0.02 ± 0.00 a	0.02 ± 0.00 bc	0.02 ± 0.00 bcde	0.02 ± 0.00 adf	0.02 ± 0.00 cde	0.02 ± 0.00 cdef	0.02 ± 0.00 bce	0.02 ± 0.00 b	0.02 ± 0.00 cde	0.03 ± 0.00 cdef	0.02 ± 0.00 adef	0.02 ± 0.00 af
	**20**	ab	0.02 ± 0.00 a	0.02 ± 0.00 ab	0.02 ± 0.00 bc	0.02 ± 0.00 cd	0.02 ± 0.00 cd	0.02 ± 0.00 de	0.02 ± 0.00 abc	0.02 ± 0.00 de	0.02 ± 0.00 e	0.02 ± 0.00 f	0.01 ± 0.00 g	0.01 ± 0.00 g
	**37**	a	0.02 ± 0.00 b	0.02 ± 0.00 b	0.01 ± 0.00 c	0.01 ± 0.00 de	0.01 ± 0.00 c	0.01 ± 0.00 d	0.01 ± 0.00 e	0.00 ± 0.00 f	0.00 ± 0.00 f	0.00 ± 0.00 f	0.00 ± 0.00 g	0.00 ± 0.00 g
	**50**	a	0.01 ± 0.00 b	0.01 ± 0.00 c	0.01 ± 0.00 d	0.00 ± 0.00 e	0.00 ± 0.00 d	0.00 ± 0.00 g	0.00 ± 0.00 f	0.00 ± 0.00 f	0.00 ± 0.00 f	-	-	-
Lutein dilinoleate	**-32**	0.00 ± 0.00 a	-	-	-	-	-	-	-	-	-	-	-	-
	**6**		0.00 ± 0.00 a	0.00 ± 0.00 a	0.00 ± 0.00 a	0.00 ± 0.00 a	0.00 ± 0.00 a	0.00 ± 0.00 a	0.00 ± 0.00 a	0.01 ± 0.00 b	0.01 ± 0.00 c	0.01 ± 0.00 d	0.00 ± 0.00 a	0.01 ± 0.00 c
	**20**		0.00 ± 0.00 a	0.00 ± 0.00 a	0.00 ± 0.00 a	0.01 ± 0.00 b	0.02 ± 0.00 b	0.03 ± 0.00 c	0.03 ± 0.00 d	0.03 ± 0.00 d	0.03 ± 0.00 d	0.05 ± 0.00 e	0.04 ± 0.00 f	0.05 ± 0.00 e
	**37**		0.01 ± 0.00 b	0.03 ± 0.00 c	0.05 ± 0.00 d	0.06 ± 0.00 de	0.08 ± 0.01 f	0.07 ± 0.00 fg	0.07 ± 0.00 fg	0.06 ± 0.00 eg	0.06 ± 0.00 de	0.05 ± 0.00 de	0.04 ± 0.00 h	0.03 ± 0.00 h
	**50**		0.06 ± 0.00 b	0.06 ± 0.00 b	0.05 ± 0.00 c	0.04 ± 0.00 d	0.03 ± 0.00 e	0.03 ± 0.00 f	0.02 ± 0.00 e	0.02 ± 0.00 e	0.00 ± 0.00 a	-	-	-

Lutein 3’-*O*-linoleate-3-*O*-palmitate and Lutein 3’-*O*-palmitate-3-*O*-linoleate	**-32**	0.00 ± 0.00 a	-	-	-	-	-	-	-	-	-	-	-	-
	**6**		0.00 ± 0.00 a	0.00 ± 0.00 a	0.00 ± 0.00 a	0.00 ± 0.00 a	0.00 ± 0.00 a	0.00 ± 0.00 a	0.00 ± 0.00 a	0.00 ± 0.00 a	0.00 ± 0.00 b	0.00 ± 0.00 c	0.00 ± 0.00 d	0.00 ± 0.00 c
	**20**		0.00 ± 0.00 a	0.00 ± 0.00 a	0.00 ± 0.00 a	0.01 ± 0.00 b	0.01 ± 0.00 b	0.01 ± 0.00 c	0.02 ± 0.00 d	0.02 ± 0.00 d	0.02 ± 0.00 d	0.02 ± 0.00 e	0.02 ± 0.00 f	0.02 ± 0.00 e
	**37**		0.01 ± 0.00 b	0.02 ± 0.00 c	0.04 ± 0.00 d	0.04 ± 0.00 d	0.05 ± 0.00 e	0.05 ± 0.00 f	0.05 ± 0.00 f	0.04 ± 0.00 d	0.03 ± 0.00 d	0.03 ± 0.00 d	0.02 ± 0.00 c	0.02 ± 0.00 c
	**50**		0.05 ± 0.00 b	0.05 ± 0.00 c	0.04 ± 0.00 c	0.03 ± 0.00 d	0.02 ± 0.00 e	0.02 ± 0.00 f	0.01 ± 0.00 e	0.01 ± 0.00 e	0.00 ± 0.00 a	-	-	-
Lutein dipalmitate	**-32**	0.00 ± 0.00 a	-	-	-	-		-	-	-	-	-	-	-
	**6**		-	-	-	-	-	-	-	-	-	-	-	-
	**20**		0.00 ± 0.00 a	0.00 ± 0.00 a	0.00 ± 0.00 a	0.00 ± 0.00 a	0.00 ± 0.00 a	0.00 ± 0.00 a	0.00 ± 0.00 bc	0.00 ± 0.00 bc	0.00 ± 0.00 b	0.00 ± 0.00 d	0.00 ± 0.00 cd	0.00 ± 0.00 bc
	**37**		0.00 ± 0.00 b	0.00 ± 0.00 bc	0.01 ± 0.00 def	0.01 ± 0.00 dgh	0.01 ± 0.00 i	0.01 ± 0.00 gi	0.01 ± 0.00 ghi	0.01 ± 0.00 de	0.01 ± 0.00 deh	0.01 ± 0.00 dh	0.01 ± 0.00 cef	0.00 ± 0.00 cf
	**50**		0.01 ± 0.00 b	0.01 ± 0.00 bc	0.01 ± 0.00 c	0.01 ± 0.00 d	0.01 ± 0.00 e	0.01 ± 0.00 f	0.00 ± 0.00 ef	0.00 ± 0.00 ef	0.00 ± 0.00 a	-	-	-
**Monoesters and Regioisomers ratios**														
Lutein monopalmitate/ Lutein monolinoleate	**-32**	0.29	0.27	0.29	0.22	0.18	0.20	0.29	0.29	0.31	0.26	0.27	0.27	0.28
	**6**		0.69	0.73	0.65	0.65	0.57	0.60	0.56	0.57	0.51	0.47	0.48	0.46
	**20**		0.61	0.65	0.59	0.55	0.58	0.55	0.58	0.56	0.56	0.53	0.53	0.51
	**37**		0.67	0.67	0.63	0.59	0.62	0.61	0.61	0.59	0.60	0.63	0.65	0.62
	**50**		0.67	0.60	0.62	0.46	0.37	0.42	0.34	0.39	0.00	0.00	0.00	0.00
Lutein 3-*O*-linoleate /Lutein 3’-*O*-linoleate	**-32**	1.04	1.09	0.96	1.03	0.96	1.12	1.03	1.30	1.20	1.19	1.11	1.06	1.09

	**6**		1.09	1.10	1.11	1.15	1.20	1.23	1.19	1.11	1.12	1.08	1.06	1.08
	**20**		1.14	1.08	1.02	1.11	1.03	0.85	0.84	0.83	0.88	0.79	0.75	0.75
	**37**		1.00	0.97	0.87	0.79	0.75	0.73	0.74	0.68	0.66	0.59	0.50	0.68
	**50**		0.77	0.83	1.06	1.26	1.38	1.11	1.09	0.76	0.49	0.00	0.00	0.00
Lutein 3-*O*-palmitate /Lutein 3’-*O*-palmitate	**-32**	1.25	1.24	1.22	1.93	1.95	1.65	1.20	1.33	1.39	1.30	1.29	1.10	1.25
	**6**		4.00	3.49	3.07	3.11	2.47	2.30	2.25	2.03	1.84	1.76	1.78	1.77
	**20**		2.40	1.40	1.05	0.93	0.90	0.76	0.76	0.69	0.71	0.65	0.67	0.61
	**37**		0.90	0.78	0.71	0.62	0.61	0.61	0.63	0.60	0.61	0.65	0.62	0.54
	**50**		0.62	0.68	0.78	1.21	1.05	0.52	0.67	0.70	0.00	0.00	0.00	0.00

[1]Data are the mean ± standard error (n=5 for the starting sample, n=3 for the rest of the samples). Different letters within the same line (for each pigment) indicate significant differences ($p<0.05$) determined by the Duncan test.
